# Biogenic engineered zinc oxide nanoparticle for sulfur black dye removal from contaminated wastewater: comparative optimization, simulation modeling, and isotherms

**DOI:** 10.1080/21655979.2024.2325721

**Published:** 2024-03-11

**Authors:** Sangita Yadav, Subhash Chander, Asha Gupta, Navish Kataria, Kuan Shiong Khoo

**Affiliations:** a Department of Environmental Science and Engineering, Guru Jambheshwar University of Science & Technology, Hisar, Haryana, India; b Department of Environmental Science and Engineering, J. C. Bose University of Science and Technology, YMCA, Faridabad, Haryana, India; c Department of Chemical Engineering and Materials Science, Yuan Ze University, Taoyuan, Taiwan; d Centre for Herbal Pharmacology and Environmental Sustainability, Chettinad Hospital and Research Institute, Chettinad Academy of Research and Education, Kelambakkam, Tamil Nadu, India

**Keywords:** Nanoparticles, zinc oxide, biogenic fabrication, sulfur black dye, removal efficiency

## Abstract

This research work aimed to isolate and culture the bacterium *Bacillus paramycoides* for biogenic fabrication of zinc oxide nanoparticles, specifically ZnO and ZnO-ME nanoparticles (nanoparticles fabricated from bacterial extracts only – ZnO, and from bacterial cell mass including extract – ZnO-ME). SEM investigation revealed the spherical-shaped NPs with 22.33 and 39 nm in size for ZnO and ZnO-ME, respectively. The Brunauer, Emmett, and Teller (BET) studies revealed mesoporous structure with pore diameters of 13.839 and 13.88 nm and surface area of 7.617 and 33.635 m^2^/gm for ZnO and ZnO-ME, respectively. Various parameters for the adsorption of sulfur black dye onto both ZnO and ZnO-ME were screened and optimized using Plackett–Burman Design (PBD), Full Factorial Design (FFD) and Central Composite Design (CCD). The results of the optimization modeling study revealed that FFD yielded the most predictable and best-fitting results among all the models studied, with *R*^2^ values of 0.998 for ZnO and 0.993 for ZnO-ME. Notably, ZnO-ME exhibited a greater dye removal efficiency 80% than ZnO i.e., 71%, it may be due to the presence of amorphous carbon on the surface of ZnO-ME. Among the various isothermal models, the Freundlich model displayed the strongest correlation with the dye removal data, confirming the multilayer adsorption of dye on both nanoparticles and supporting physisorption. Therefore, ZnO and ZnO-ME nanoparticles have been proven as potential tools for mitigating environmental impacts associated with dye-containing wastewater.

## 1. Introduction

Every government must ensure that its citizens have the access to clean, safe drinking water, yet as economies develop, many nations experience a depletion of their natural resources, especially freshwater [1,2]. Nowadays, water resources are continually contaminated by a diverse array of pollutants, for instance, dyes, micropollutants, pesticides, pharmaceuticals and heavy metals, creating a significant global environmental problem [3]. This contamination is particularly prevalent in aquatic environments due to the widespread use of synthetic dyes in diverse industrial applications like plastics, paper, antiseptics, cosmetics, food, textiles, and leather [4]. Among these dyes, sulfur dyes stand out as one of the most commonly used in the textile industry because of their affordability and capacity to produce darker, more subdued hues of color, including green, dark blue, black, brown, and olive [5].

However, the exposure and transport of wastewater containing these dyes from textile production can pose significant risks to aquatic life and plant ecosystems. Acute exposure to this toxic dye can result in various health issues in humans, including higher heart rate, nausea, shock, cyanosis, jaundice, quadriplegia, and tissue necrosis. Therefore, before discharging industrial effluents into the environment, it is imperative to remove color from effluent. Numerous methods such as solvent extraction, adsorption, membrane filtration, photocatalytic degradation using metal oxide, combined electrochemical degradation process, ozonation, advanced oxidation process, and ultrasonication have been adopted to treat industrial wastewater [5,6]. Adsorption is one of these methods that has attracted particular attention because of its technological viability, versatility, and simplicity of use. Ion-exchange materials, bentonite, zeolites, and activated carbon clays are commonly used as adsorbents [7]. Their broad range of uses can be attributed to their ordered pore size, vast surface area, excellent stability, ease of production, simplicity, rapidity, specificity, sensitivity, and cost-effectiveness [8,9]

Furthermore, recent research has focused toward the fabrication of nanomaterials for wastewater treatment [10]. The implementation of metal oxide nano-adsorbents in the adsorption process has gained attraction within the scientific community, representing the next generation of water treatment nanotechnology [12]. However, traditional approaches to producing these adsorbents, such as pyrolysis and chemical synthesis, have some limitations, including high preparation costs, hazardous chemicals, and high energy demands. Therefore, the biogenic synthesis of nanomaterials has been widely studied as it offers a more environmentally friendly and economical approach. Highly effective bioreducing, biocapping, and biostabilizing agents that are derived from biological sources are essential for producing high-quality yields of nanomaterials [11]. For instance, the biosynthesis of nanoparticle using *Gelidium amasii* (marine red alga) has exhibited antibacterial agents especially against prominent microfouling bacteria [12]. While a recent study used *Purpureocillium lilacinum* (filamentous fungus) for the biosynthesis of CuO NPs exhibited photocatalysis of navy blue and safranin dye of 57.5% and 63% removal, respectively [13].

This research work focuses on the bacterial synthesis, characterization, and application of ZnO (nanoparticles fabricated from bacterial extracts) and ZnO-ME (bacterial cell mass) nanoparticles for the adsorption of sulfur black dye from synthetic solutions. *Bacillus paramycoides* bacteria was isolated and cultivated to produce these nanoparticles, which were subsequently analyzed using techniques such as zeta potential, X-ray diffraction (XRD), Fourier-transform infrared spectroscopy (FTIR), UV–visible spectrophotometry (UV–VIS), field emission scanning electron microscopy (FE-SEM), energy-dispersive X-ray spectroscopy (EDX), and Brunauer–Emmett–Teller (BET) analysis. The optimization of various factors affecting the adsorption efficiency of the synthesized nanoparticles was performed using Plackett–Burman Design (PBD), Full Factorial Design (FFD), and Central Composite Design (CCD). Lastly, isotherm models were employed to gain insights into the adsorption process.

## 2. Materials and methods

### 2.1. Chemicals

Sulfur black dye, raw wastewater samples before treatment, and soil and sludge samples were obtained from RSWM Limited (a unit of LNJ denim), the textile industry located in Banswara, Rajasthan, India, with permission obtained from the landowner and local authorities. The wastewater was collected into an airtight bottle and placed in an ice-packed container. It was transported into the laboratory and transferred into the refrigerator at 5°C. The chemicals employed in this study, zinc sulfate heptahydrate (99%, CAS No.: 7446-20-0), absolute ethanol (>99%, CAS No.: 64-17-5), sodium hydroxide (98%, CAS No.: 1310-73-2), and hydrochloric acid (37%, CAS No.: 7647-01-0) were analytical grades and used as such supplied by Merck (Sigma-Aldrich, Chemicals Pvt. Ltd., Bangalore, India). Analytical-grade nutrient broth (SKU: M002) and agar (SKU: PCT0901) were used to perform the experiments and were purchased from Himedia Laboratories Pvt. Ltd., Mumbai, India.

### 2.2. Bacterial isolation and screening

Differential media was used for the isolation of bacterial species from the samples of wastewater, soil, and sludge. Serial dilution was performed for all the collected samples, with dilutions from 10^−1^ to 10^−10^. The prepared dilution and undiluted samples were then spread onto nutrient agar plates supplemented with one mM metal salt (zinc sulfate) and 100 mg/L sulfur black dye. The experiments were performed in triplicates. The isolation of bacterial strain was achieved through repeated streaking process, and the bacterial strain was subsequently stored for further investigation and analysis.

### 2.3. Molecular characterisation and phylogenetic analysis

Molecular characterization of isolated bacterial strain was performed by 16S rRNA technique. Using the primers fD1 (5’-AGAGTTTGATCCTGGCTCAG-3“) and rD1 (5”-AAGGAGGTGATCCAGCC-3’), the 16S rRNA gene was amplified [14]. The Microbial Culture Collection at the National Center for Cell Science in Pune, Maharashtra, India, sequenced the amplified 16S rRNA gene product on a commercial scale. The received sequence was aligned using MEGA 11 with homologous sequences discovered through NCBI’s BLASTn similarity searching. Using the neighbor-joining method (NJ method) of the MEGA 11 software, a phylogenetic tree was constructed to accurately determine the phylogenetic identity of the strain.

### 2.4. Biosynthesis and characterization of ZnO and ZnO-ME nanoparticles

**Figure 1.**
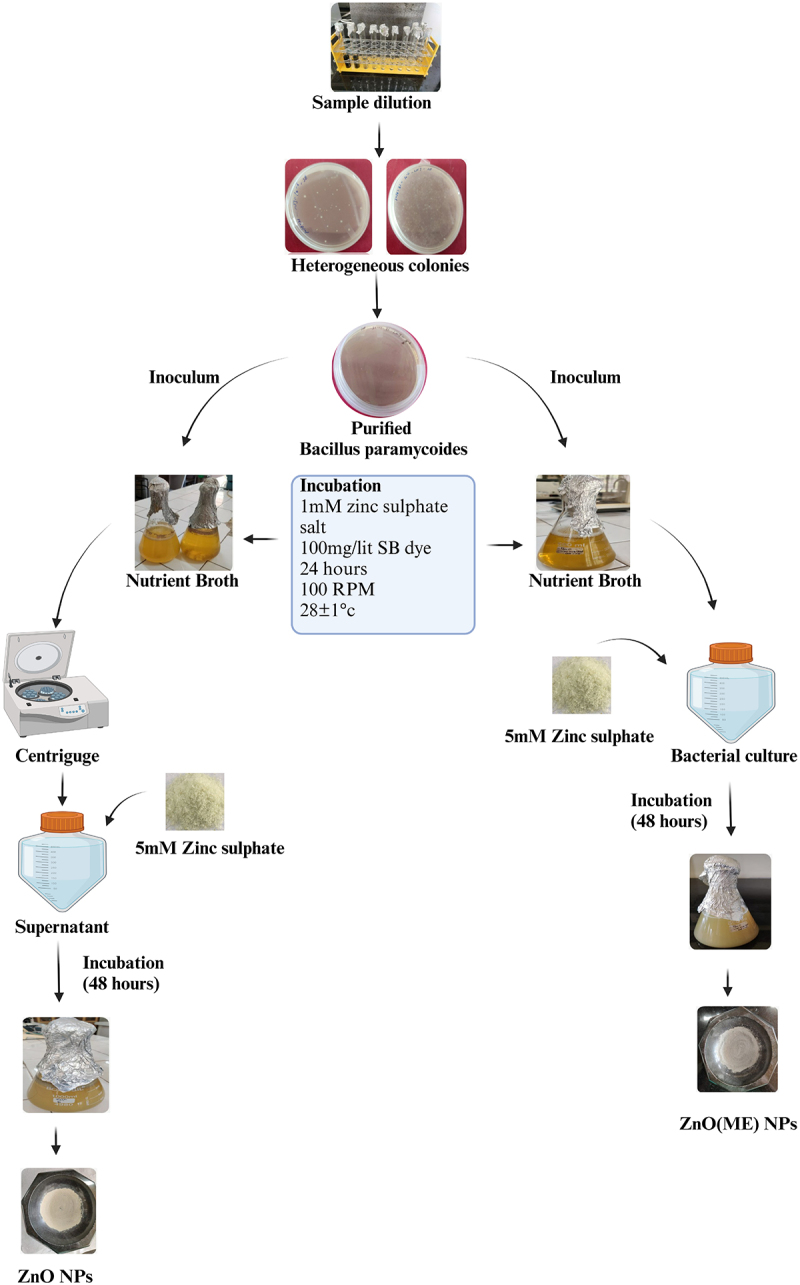
Synthesis of zinc oxide nanoparticles via isolated bacterial strain.

The identified bacteria were cultivated at 28 ± 1°C and 100 rpm for 24 h in a nutrient broth medium supplemented with 1 mM zinc sulfate and 100 mg/l of sulfur black dye. For the synthesis of ZnO-ME (bacterial cell mass including extract), 5 mM zinc sulfate was added to the bacterial culture after 24 h of incubation. The mixture was then incubated under the same conditions for an additional 48 h. On the other hand, the bacterial culture supernatant was extracted for the synthesis of ZnO (nanoparticles fabricated from bacterial extracts) by centrifugation at 6000 rpm for 5 min. The supernatant was then mixed with 5 mM zinc sulfate, and the mixture was incubated for an additional 48 h under the same conditions. The observation of a change in the color of the solution served to confirm the biological synthesis of ZnO and ZnO-ME nanoparticles, as shown in Figure 1. Organic/biomolecules are used as ligands (reducing and capping) in the reduction of metal ions to create metal NPs [15]. Following synthesis, the nanomaterials were separated from both reaction mixtures by centrifuging them at 6000 rpm for 5 min, with and without bacterial cell mass. The obtained nanomaterials were subjected to an oven at 150°C for 72 h and then using a pestle and mortar, crushed into a fine powder. The surface charge, wettability, and geometry of nanochannels can be precisely regulated in response to external stimulus, such as pH, temperature [16]. The final ZnO and ZnO-ME nanoparticles were stored in plastic bottles in a desiccator for use in subsequent dye removal studies.

The physio-chemical characteristics of the nanoparticles were thoroughly investigated through zeta-potential, XRD, FTIR, UV–VIS, FE-SEM, EDX, and BET analysis. In order to analyze the absorption spectra of ZnO and ZnO-ME, a UV–VIS spectrophotometer was utilized. The colloidal solution of the synthesized NPs was used to measure the zeta potential using the Zetasizer (Malvern Instruments) and dynamic light scattering methods. FE-SEM imaging was used to determine nanoparticle surface morphology and size, while ESEM and EDX examination involved casting gold-coated solutions onto glass slides. In order to identify surface functional groups, FTIR analysis was carried out in the range 400–4000 cm^−1^ and for the analysis, a dried powder sample was mixed with KBr. XRD analysis was used to determine the nanoparticles’ crystalline structure, which involved scanning in 2 theta degrees ranging from 20° to 80°. In the XRD analysis, the dried, powdered material was employed. CuKα radiation was used to study the nanoparticles, using 40 kV voltage and 40 mA of current. The specific surface area values were calculated using Autosorb IQ_2_ Quantachrome equipment and the BET (Brunauer–Emmett–Teller) mathematical method employing cycles of N_2_ adsorption and desorption.

### 2.5. Adsorption experiments

At room temperature, experiments were conducted in 100 mL conical flasks in an incubator shaker (Scigenics Biotech, Orbitek) with a 160-rpm agitation speed. Sulfur black dye ($C_{6}H_{3}{\left[\mathrm{OH}\right]}^{-}[NO_{2}{]}_{2}^{+}$) due to presence of negative charge on its surface precipitates out at higher pH that’s why in the current study solution’s pH was held constant at 7.0. Lim et al. (2021) showed the discolouration and precipitation of methyl blue and methyl orange dye at higher pH value [17]. Room temperature is used in this study to make the adsorption process more cost-effective and reliable. The contact period ranged from 60 to 180 min, while the initial sulfur black dye concentration ranged from 10 to 100 mg/L in this research. Similarly, the amount of adsorbent used varied from 5 to 25 mg for each 20 mL of synthetic sulfur black dye solution. To assess the efficiency of dye’s adsorption onto the ZnO and ZnO-ME nanomaterials, the initial and final concentrations of sulfur black dye were measured using a UV–VIS spectrophotometer.

### 2.6. Experimental design

This study examined the impact of various independent variables on the removal of sulfur black dye from simulated water, including initial dye concentration (10–100 mg/L), contact time (60–80 min), and ZnO or ZnO-ME dosage (5–25 mg). Minitab software version 21 was used for PBD and FFD statistical analysis, and Research Design Expert version 13 was utilized for CCD statistical analysis. To determine how well these models fit the experimental data, the variance analysis (ANOVA) was used. The Fischer (*F*-test) test with a 95% confidence interval was used to assess the models’ significance as well as the interactions between the variables and the related responses.

#### 2.6.1. Central Composite Design (CCD)

The CCD analyzes the relationships between process variables and their corresponding responses. It is useful for estimating second-order polynomial equations over a larger portion of the design space. In CCD design, the five-level fractional factorial consisted of two axial designs, two central designs, and a two-level factorial design is included. To improve the measurement of reproductive capacity and the lack of fit in the models, the central point is repeated. Additionally, CCD designs feature orthogonality and rotatability characteristics, which facilitate curvature representation in 3D charts [18]. (Equation 1) of CCD model is as follows:

(1) $$Y_{\mathrm{pred}}=\beta_{0}+\sum_{i=1}^{k}\beta_{i}x_{i}+\sum_{i=1}^{k}\sum_{j=i+1}^{k}\beta_{\mathrm{ij}}x_{i}x_{j}+\sum_{i=1}^{k}\beta_{\mathrm{ii}}X_{i}^{2}$$

In this model equation, *x*_*i*_ and *x*_*j*_ are coded values of independent variables, a model constant (*β*_*0*_), the expected response (*Y*), the number of independent variables (*k*), and regression coefficients for linear, quadratic, and interaction terms (*β*_*i*_, *β*_*ii*_, *β*_*ij*_).

#### 2.6.2. Plackett–Burman Design (PBD)

PBD was employed to determine the significance of the aspects under investigation. The design had various combinations of the independent elements in each run (A-C). Three levels of these parameters were examined: low (−), high (+), and center (0). The aim of this multivariate regression analysis was to identify the crucial variables in influencing the experimental response. PBD was established using the linear model expressed as (Equation 2):

(2) Y = βo +∑4 i=1 βiXi

where the regression coefficients for intercept and linear expression are *βo* and *βi*, respectively; *X*_*i*_ represents the coded independent factors and *Y* represents the experimental response [19].

#### 2.6.3. Full Factorial Design (FFD)

Multiple regression analysis was used to determine the relationship between the response variable and the three factors. The model equation is expressed as follows:

(3) $$R\;\left(\%\right)\;=X_{0}+X_{1}A\;+X_{2}B\;+X_{3}C\;+X_{4}\mathrm{AB}\;+X_{5}\mathrm{AC}\;+X_{6}\mathrm{BC}\;+X_{7}\mathrm{ABC}$$

where initial sulfur black dye concentration, contact time, and ZnO or ZnO-ME amount are denoted as *A, B*, and *C*, respectively, with a response of *R*, global mean *X*_*0*_, and remaining regression coefficients *X*_*i*_ [20]. The full factorial design with two or more levels is considered as most effective design that may be used to screen numerous parameters, identify the critical components, and determine their suitable levels.

### 2.7. Determination of removal efficiency and adsorption capacity

Using UV–VIS spectroscopy at a wavelength of 600 nm, the initial and residual amounts of sulfur black dye were assessed. The standard curve of absorbance against a stock solution of sulfur black dye with concentrations ranging from 10 to 100 mg/L was used to measure the sulfur black dye concentration (refer to Supplementary Information (SI)). The sulfur black dye’s adsorption capacity (mg/g) and removal effectiveness (percent) were expressed as follows:

(4) $$\mathrm{Removal}\;\;\mathrm{efficiency}\left(\%\right)=\frac{\left(C_{o}-C_{e}\right)}{C_{o}}\times 100$$

(5) $$\mathrm{Adsorption}\;\;\mathrm{capacity}\left(q_{e}\right)=\frac{\left(C_{o}-C_{e}\right)*V}{m}$$

where *m (g)* is the mass of the ZnO and ZnO-ME, *V (L)* is the working solution volume used in the experiments, $C_{o}$ (mg/L) and $C_{e}$ (mg/L) is the initial concentration, and the final concentration in the solution, respectively [21].

### 2.8. Adsorption isotherm

Adsorption isotherms more accurately represent the equilibrium behavior by graphing the amounts of adsorbent adsorbed per unit amount of adsorbent against the corresponding equilibrium concentration in the solution phase. The equations of the Langmuir (Equation 6), Freundlich (Equation 7) and Temkin (Equation 8) isotherm adsorption models are given below:

(6) $$\frac{C_{e}}{q_{e}}=\frac{1}{Q_{o}}C_{e}+\frac{1}{Q_{o}b}$$

The values of *Q*_*0*_ and *b* were determined using the slope and intercept of the Langmuir plot of *C*_*e*_ versus *C*_*e*_/*q*_*e*_, and a dimensionless constant called separation factors (*R*_*L*_) was used to test the favorability of the process [22].

(7) $$\mathrm{Log}\;q_{e}=\frac{1}{n}\mathrm{Log}\;C_{e}+\mathrm{Log}\;K_{f}$$

The *K*_*f*_ and *1*/*n* values were determined by plotting the graph between log *q*_*e*_ and log *C*_*e*_. High *K*_*f*_ and high ‘*n*’ values indicate high sorption throughout the concentration range, while low *K*_*f*_ and high ‘*n*’ values indicate low sorption. Low ‘*n*’ values indicate high sorption at strong solute concentration. Beneficial sorption occurs at *n* values between 1 and 10 [23].

(8) $$q_{e}=\frac{\mathrm{RT}}{b}\mathrm{Log}\;C_{e}+\frac{\mathrm{RT}}{b}\mathrm{Log}\;a$$

Where *q*_*e*_ is the quantity of adsorbate sorbed per unit amount of sorbent (mg/g); *C*_*e*_ is the concentration of sorbate at equilibrium (mg/L); *R* is the gas constant (0.0083 kJ/mol K); *T* is the temperature (K); *b* is the Temkin constant related to heat of sorption (kJ/mol); *a* is the Temkin isotherm constant called equilibrium binding constant (L/g) corresponding to the maximum binding energy [24]. A plot of *q*_*e*_ versus log *C*_*e*_ enables the determination of the isotherm constants *a* and *b*.

## 3. Result and discussion

This study investigates the synthesis, characterization, and application of ZnO and ZnO-ME nanoparticles from *Bacillus paramycoides* bacteria for the adsorption of sulfur black dye from synthetic solutions. Techniques including zeta potential, XRD, FTIR, UV–VIS, FE-SEM, EDX, and BET analysis were used. The adsorption efficiency was optimized using Plackett–Burman Design, Full Factorial Design, and Central Composite Design, and isotherm models were used for further understanding.

### 3.1. Molecular characterisation and phylogenetic analysis

**Figure 2.**
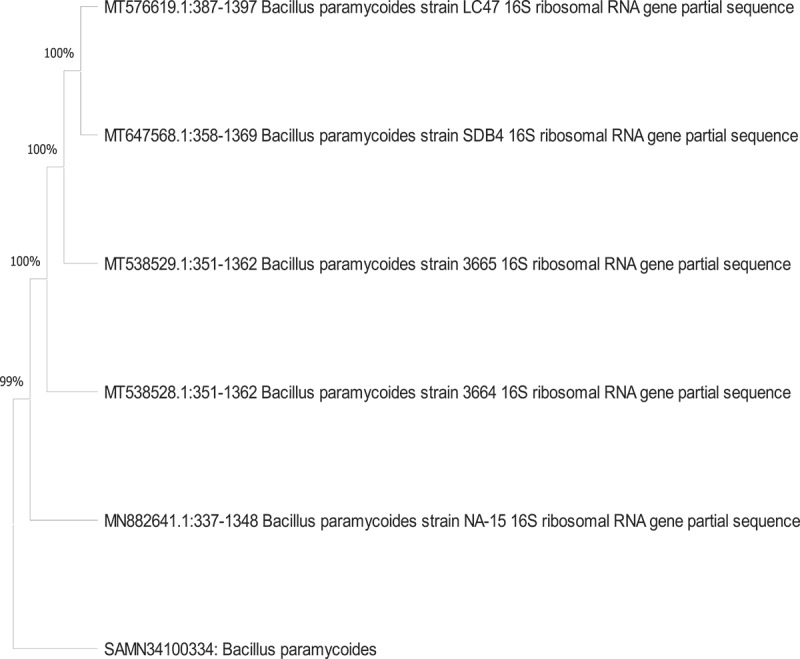
Phylogenetic tree of isolated *bacillus paramycoides*.

Bacterial isolates were extracted using differential media from the textile wastewater, sludge, and soil samples. Using the NCBI’s BLAST program, the isolated strain’s 16S rRNA gene sequence was compared to previously reported 16S rRNA sequences of bacteria and archaea and revealed similarities with numerous *Bacillus* species, as shown in Figure 2. The isolated bacterial strain from the wastewater sample was identified as *Bacillus paramycoides*. Furthermore, the 16S rDNA gene sequence is linked to the gene bank (https://www.ncbi.nlm.nih.gov). The accession number for strains was obtained as SAMN341003344.

### 3.2. Characterization of ZnO and ZnO-ME nanoparticles

#### 3.2.1. Zeta Potential (ZP)

The zeta potential (ζ-potential) of the suspended nanomaterials was measured via dynamic light scattering by means of a Zetasizer (Malvern Instruments, UK). The pH of the suspension was neutral while conducting zeta potential experiment as further in this study the adsorption experiments were carried out at neutral pH. ZnO and ZnO-ME synthesized through bacterial sources displayed a zeta potential value of −22.4and −17.2 mV, respectively (refer to Supplementary Information, Fig SI-2). The correlation of ZP data with colloid stability is a widely used application in nanoparticle characterization. According to established classifications, nanomaterial dispersions with ZP values between ± 0 and ±10 mV, ±10 and ±20 mV, ±20 and ±30 mV, and > ±30 mV are, respectively, categorized as highly unstable, reasonably stable, moderately stable, and highly stable [25]. ZnO nanoparticles are moderately stable, whereas ZnO-ME nanoparticles are reasonably stable, according to zeta potential measurements. These zinc nanoparticles are coated with negatively charged biomolecules, as indicated by the negative value [26].

#### 3.2.2. FTIR analysis

**Figure 3.**
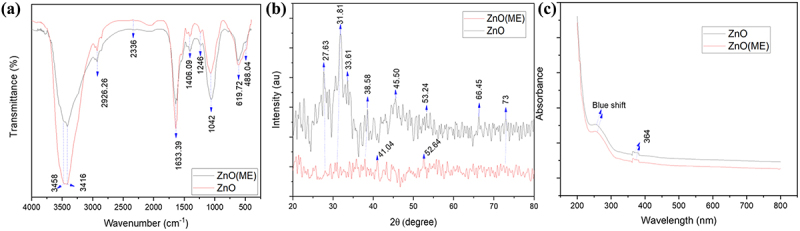
Characterization of ZnO and ZnO-ME. (a) FT-IR of ZnO and ZnO-ME (before dye adsorption), (b) XRD of ZnO and ZnO-ME and (c) UV–VIS spectrum of ZnO and ZnO-ME.

The FTIR spectra of the nanoparticles are depicted in Figure 3a. In FTIR spectra of the synthesized zinc oxide nanoparticles (ZnO), a strong peak was observed at 484.04 and 619.72 cm^−1^, suggesting the biogenic synthesis of zinc oxide nanoparticles. Metal oxides, including zinc oxide, have vibrational peaks between 400 and 600 cm^−1^ [27]. The presence of water molecules can be inferred from the broadband that occurred between 3416 and 3458 cm^−1^ since the O–H stretch is associated with this peak. The –C=C stretching vibration of alkynes is responsible for the two prominent peaks at 2336 cm^−1^. The C–H and HO–C=O groups may bend symmetrically and asymmetrically to produce the spectral peak at 2926.26 cm^−1^ [28]. The N-H stretching vibration of amine groups on the sample’s surface is suggested by high absorption peaks at 1633.39 cm^−1^. Absorption peak at 1246 cm^−1^ due to C-N stretching of anime group. In-plane C–H bending is responsible for the absorption peaks at 1042 and 1406.09 cm^−1^ [29]. Similar peaks were observed in ZnO-ME; however, they were less intense compared to ZnO.

#### 3.2.3. XRD spectrum analysis

Figure 3b illustrates the XRD pattern of both the synthesized ZnO and ZnO-ME nanoparticles. The XRD peaks are revealed at 2 theta angles of 27.63°, 31.81°, 33.61°, and 38.58°, corresponding to the (2 0 1), (1 0 0), (0 0 2), and (1 0 1) planes of ZnO, respectively. Additionally, there are several diffraction peaks at 2 theta angles, including 45.50°, 52.64°, 53.24°, 66.45°, and 73°, which, respectively, correspond to the (1 0 2), (1 1 0), (2 0 1), and (1 1 2) planes of ZnO. These diffraction peaks of the ZnO are consistent with the data provided for standard ZnO in the Joint Committee on Powder Diffraction Standards (JCPDS). The noisy narrow nature of these distinctive peaks indicates that the produced nanoparticles are amorphous [30]. The XRD pattern of ZnO-ME, on the other hand, exhibits an amorphous nature as the peaks are not sharp. Peaks at 2 theta angles 31.761°, 34.424°, 47.541° are reported by Rajabairavi et al. (2017) for zinc oxide nanoparticles [31]. Ahmad and Kalra (2020) discovered diffraction peaks at 2 theta values of 31.79°, 34.46°, 36.25°, 47.47°, 56.61°, and 66.43°, which are comparable to the results of the current investigation [29]. In case of ZnO-ME nanomaterials, other noisy peaks at 2*θ* between 20° and 24° revel the presence of amorphous carbon from bacterial cell mass [32].

#### 3.2.4. UV–VIS analysis

UV–VIS spectroscopy was used to describe the structural characteristics of ZnO and ZnO-ME nanomaterials. In the UV region, absorption peaks were seen for the ZnO. Both nanomaterials exhibited an average absorption wavelength of 364 nm, as shown in Figure 3c. According to Vijayakumar et al. (2018) and Shanavas et al. (2019), the absorption bands of zinc oxide nanoparticles typically fall between 330 and 370 nm [28,33]. According to Vaseem et al. (2012), the quantum confinement effect of ZnO nanoparticles led to a blue-shifted near band-edge UV absorption at 340 nm [34]. When compared to the bulk absorption edge, which at ambient temperature appears at 373 nm, a larger excitonic absorption pattern at about 345 nm is blue-shifted by roughly 30 nm [35]. The absorption peak underwent a blue shift and was found at about 229 nm which accordance with the smaller nanoparticle size [36]. The ZnO’s energy gap (*Eg*) was calculated using (Equation 9):

(9) $$\mathrm{Eg}=\frac{\mathrm{hc}}{\lambda }$$

Where the wavelength of light is *λ*, the Planck constant is *h*, and the speed of light is *c*. The energy gap of the nanomaterials,’ denoted as *Eg*, was estimated to be 3.4 eV.

#### 3.2.5. FE-SEM, EDX, and BET analysis

**Figure 4.**
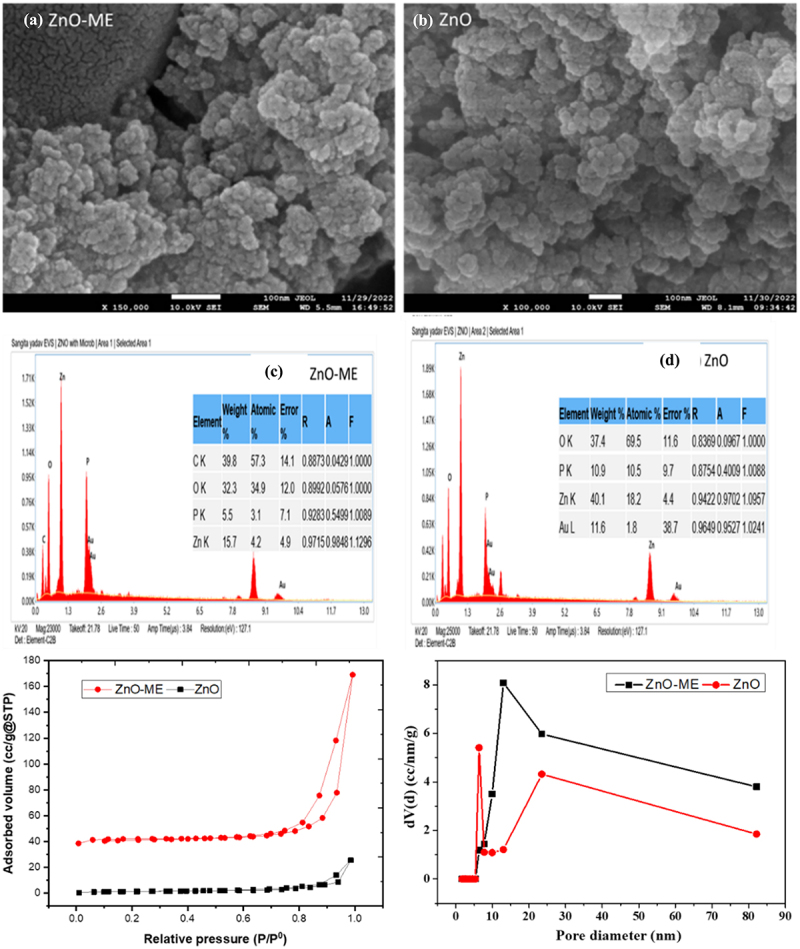
(a,b) surface morphology analysis, (c,d) EDX spectrum and (e,f) nitrogen adsorption–desorption curve and pore size distribution for ZnO-ME and ZnO.

Figure 4a,b shows the FE-SEM images that illustrate the surface characteristics of biogenic ZnO and ZnO-ME, in which spherical-shaped aggregates of zinc oxide were formed. Previous research reported by Singh et al.. (2010) used *Escherichia coli* for the biosynthesis of aggregated micrometer copper oxide nanoparticles [37]. This spherical shape of ZnO has also been observed in other biological synthesis methods using *Pseudomonas fluorescens*, where ZnO was found to aggregate on the surface of bacterial cells [38]. The spherical ZnO and ZnO-ME nanoparticles’ measured diameters were discovered to be approximately 22.33 and 39 nm, respectively, using imageJ software.

Figure 4c,d show the findings of analyzing the elemental composition of zinc oxide nanoparticles using EDX spectroscopy. The peaks at various keV levels are clearly visible in the EDX spectrum, which suggests that the adsorbent contains zinc, oxygen, and carbon.

Relative pressure (P/P0) and adsorbed volume (cc/g at STP) are plotted in the N_2_ adsorption–desorption isotherms, along with the BJH pore size distribution of the ZnO-ME and ZnO nanomaterials were analyzed by BET analyzer, as shown in Figure 4e,f. The isotherm curve closely resembles a normal type IV isotherm graph, demonstrating the H3 hysteresis loop and supporting the mesoporous feature of the nanomaterials. Mesoporous zinc oxide nanoparticles were also obtained in a prior investigation by Varadavenkatesan et al. (2019) [39]. This kind of loop exhibits mono-multilayer adsorption, and it is well known that the surface area of the adsorbent has a significant impact on adsorption. The adsorption processes are facilitated by a wide surface area and porosity, which offer a large number of active sites [40]. BET analysis for nanomaterials indicated a specific surface area of 7.617 and 33.635 m^2^/g for ZnO and ZnO-ME, respectively. Additionally, the total pore volumes for ZnO, and ZnO-ME were 0.045 and 0.218 cc/g, respectively, and average pore diameters discovered to be 13.839 and 13.882 nm, respectively.

### 3.3. Optimization models for dye absorption

#### 3.3.1. Central composite design

CCD is utilized to assess the most important interdependence between variables as well as the individual effects of each variable on the dye removal efficiency. The significant correlation between each variable and their responses is expressed in (Equation 10) and (Equation 11) below:

*Removal efficiency (ZnO-ME)*

(10) Y=79.2514 + 3.54159 ∗ A + 4.62039 ∗ B + 4.53316 ∗ C

*Removal efficiency (ZnO)*

(11) Y=70.7573 + 3.49239 ∗ A + 3.12299 ∗ B + 4.41001 ∗ C + 0.625 ∗ AB + −0.125 ∗ AC + −0.125 ∗ BC

Where *Y* stands for dye removal efficiency (%), *A* stands for dose; *B* stands for dye concentration, and *C* stands for contact time between nanomaterials and sulfur black dye.

**Figure 5.**
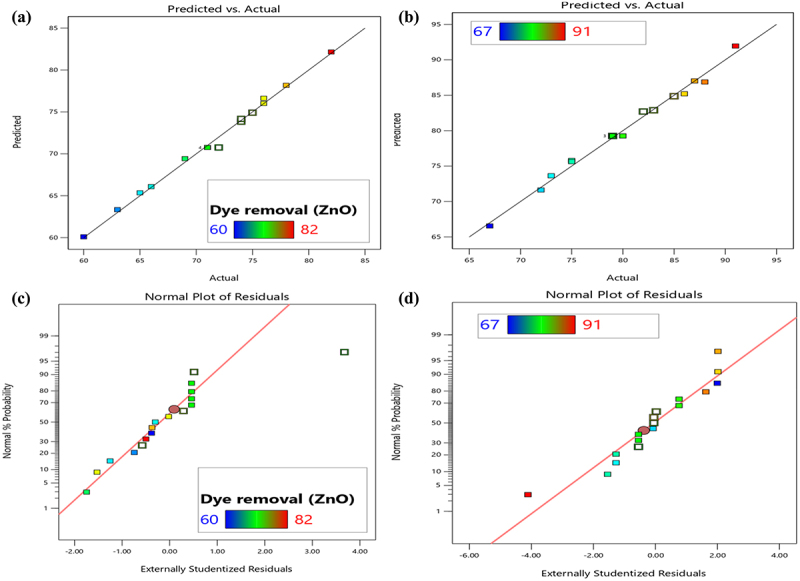
Predicted against actual values plots for (a) for ZnO and (b) for ZnO-ME and normal percentage probability plots for (c) for ZnO and (d) for ZnO-ME for sulfur black dye adsorption.

The ANOVA and model statistics summary utilized to analyze the observed experimental data for sulfur black dye adsorption are shown in Tables 1 and 2, respectively. High *F*-values for ZnO and ZnO-ME (303.79 and 431.11, respectively) and a probability *p*-value of less than 0.0001 demonstrate significance of the model. The non-significant lack of fit test was confirmed by the low *F*-value of >0.0500. Using the proper precision, standard deviation, coefficient of variance, and regression coefficient, the precision, reliability, accuracy, and standard deviation of the experimental data were assessed. The ‘Adequate precision,’ which defines the signal-to-noise ratio, has a value of 4 that is suitable for models. The relationship between standard deviation and mean was shown by the CV percent values of 0.73 for ZnO and 0.87 for ZnO-ME. If the CV is less than one, or 100%, the standard deviation is less than the mean. The ‘Adjusted *R*^2^’ was almost closer to the ‘Predicted *R*^2^,’ and the deviation between these was less than 0.02, indicating that the experimental data was very well suited and tailored by the model. The experimental results (dye adsorption %) are in perfect agreement with the model’s anticipated value, as shown by the *R*^2^ value. The linear correlation between the predicted response and actual experimental response for both nanoparticles for the percentage of sulfur black dye adsorption is shown in Figure 5. It shows the degree to which the experimental results and the theoretical distribution are comparable. It demonstrates that there is good agreement between the model predictions and the actual experimental values of sulfur black dye adsorption. The interaction of two variables and their combined effect on sulfur black dye removal is expressed in 3D model graph as shown in Fig. S3. Plots with curvature verified the degree of interaction between the independent process variables [41].

**Table 1.** Analysis of variance (ANOVA) for the adsorption of sulfur black dye using ZnO and ZnO-ME nanoparticles

ZnO												ZnO-ME								
Source	Sum of Squares		df	Mean Square	F-value		p-value				Source	Sum of Squares	df			Mean Square	F-value	p-value		
**CCD-Analysis of variance (ANOVA) for the adsorption of sulfur black dye using ZnO and ZnO-ME nanoparticles**																				
Model	499.38		6	83.23	303.79		< 0.0001		significant		Model	636.54	3			212.18	431.11	< 0.0001		significant
A-dose	130.01		1	130.01	474.54		< 0.0001				A-dose	133.7	1			133.7	271.65	< 0.0001		
B-conc.	103.96		1	103.96	379.47		< 0.0001				B-conc.	227.56	1			227.56	462.35	< 0.0001		
C-contact time	265.6		1	265.6	969.45		< 0.0001				C-contact time	280.64	1			280.64	570.21	< 0.0001		
AB	3.12		1	3.12	11.41		0.007													
AC	0.125		1	0.125	0.4563		0.5147													
BC	0.125		1	0.125	0.4563		0.5147													
Residual	2.74		10	0.274							Residual	6.4	13			0.4922				
Lack of Fit	1.94		6	0.3233	1.62		0.3342		not significant		Lack of Fit	5.2	9			0.5776	1.93	0.2758		not significant
Pure Error	0.8		4	0.2							Pure Error	1.2	4			0.3				
Cor Total	502.12		16								Cor Total	642.94	16							
ZnO-ME										ZnO										
Source		DF				Adj SS	Adj MS	F-Value	P-Value	Source			DF	Adj SS	Adj MS	F-Value			P-Value	
**PBD-Analysis of variance (ANOVA) for the adsorption of suflur black dye using ZnO and ZnO-ME nanoparticles**																				
Model		3				631.583	210.528	461.12	0	Model			3	497.583	165.861	315.97			0	
Linear		3				631.583	210.528	461.12	0	Linear			3	497.583	165.861	315.97			0	
Dose (mg)		1				126.75	126.75	277.62	0	Dose (mg)			1	154.083	154.083	293.53			0	
Conc. (mg/lit)		1				270.75	270.75	593.03	0	Conc. (mg/lit)			1	126.75	126.75	241.46			0	
Contact time (min)		1				234.083	234.083	512.72	0	Contact time (min)			1	216.75	216.75	412.91			0	
Error		9				4.109	0.457			Error			9	4.724	0.525					
Curvature		1				0.776	0.776	1.86	0.21	Curvature			1	0.058	0.058	0.1			0.761	
Lack-of-Fit		4				2.833	0.708	5.67	0.061	Lack-of-Fit			4	4.167	1.042	8.33			0.032	
Pure Error		4				0.5	0.125			Pure Error			4	0.5	0.125					
Total		12				635.692				Total			12	502.308						
ZnO-ME										ZnO										
Source		DF				Adj SS	Adj MS	F-Value	P-Value	Source				DF	Adj SS	Adj MS			F-Value	P-Value
**FFD-Analysis of variance (ANOVA) for the adsorption of suflur black dye using ZnO and ZnO-ME nanoparticles**																				
Model		3				415.375	138.458	263.73	0	Model				4	339.5	84.875			679	0
Linear		3				415.375	138.458	263.73	0	Linear				3	336.375	112.125			897	0
Dose (mg)		1				91.125	91.125	173.57	0	Dose (mg)				1	105.125	105.125			841	0
Conc. (mg/lit)		1				171.125	171.125	325.95	0	Conc. (mg/lit)				1	78.125	78.125			625	0
Contact time (min)		1				153.125	153.125	291.67	0	Contact time (min)				1	153.125	153.125			1225	0
Error		5				2.625	0.525			2-Way Interactions				1	3.125	3.125			25	0.007
Curvature		1				1.125	1.125	3	0.158	Dose (mg)*Conc. (mg/L)				1	3.125	3.125			25	0.007
Lack-of-Fit		4				1.5	0.375	*	*	Error				4	0.5	0.125				
Total		8				418				Curvature				1	0.125	0.125			1	0.391
										Lack-of-Fit				3	0.375	0.125			*	*
										Total				8	340

**Table 2.** Model summary and statistical analysis.

Model	*R*^2^	Adjusted *R*^2^	Predicted *R*^2^	Std. Dev.	Mean
CCD (ZnO)	0.9945	0.9913	0.9838	0.5234	71.41
CCD (ZnO-ME)	0.9900	0.9878	0.9814	0.7016	80.06
FFD (ZnO)	0.9985	0.9971	0.9920	0.3535	70.67
FFD (ZnO-ME)	0.9937	0.9900	0.9823	0.7245	79.00
PBD (ZnO)	0.9906	0.9875	0.9793	0.7245	70.77
PBD (ZnO-ME)	0.9935	0.9914	0.9869	0.6756	79.15

#### 3.3.2. Plackett–Burman Design (PBD)

Unbiased screening of the variables that significantly influence the sulfur black dye’s adsorption onto ZnO and ZnO-ME was done using a two-level PBD factorial design with 13 runs. The results of a three-factor experiment served as its foundation. The significance of dye removal experiment results is stated as an ANOVA, and 3D plots as shown in Table 1 and Figure S4, respectively. The surface plots of the response functions facilitate the combined impact of the investigated factors and their interaction. The correlation between the studied variables and their responses for both the nanoparticles for the sulfur black dye adsorption % are expressed as in (Equation 12) and (Equation 13) below:

(12) $$\mathrm{Removal}\;\;\mathrm{efficiency}\;\left(\mathrm{ZnO}-\mathrm{ME}\right)\;=59.640\;+\;0.3250\;\;\mathrm{Dose}\;\left(\mathrm{mg}\right)\;+\;0.10556\;\;\mathrm{Conc}.\;\left(\mathrm{mg}/L\right)\;+\;0.07361\;\;\mathrm{Contact}\;\;\mathrm{time}\;\left(\min\right)$$

(13) $$\mathrm{Removal}\;\;\mathrm{efficiency}\;\left(\mathrm{ZnO}\right)\;=\;52.922\;+\;0.3583\;\;\mathrm{Dose}\;\left(\mathrm{mg}\right)\;+\;0.07222\;\;\mathrm{Conc}.\;\left(\mathrm{mg}/L\right)\;+\;0.07083\;\;\mathrm{Contact}\;\;\mathrm{time}\;(\min$$

**Figure 6.**
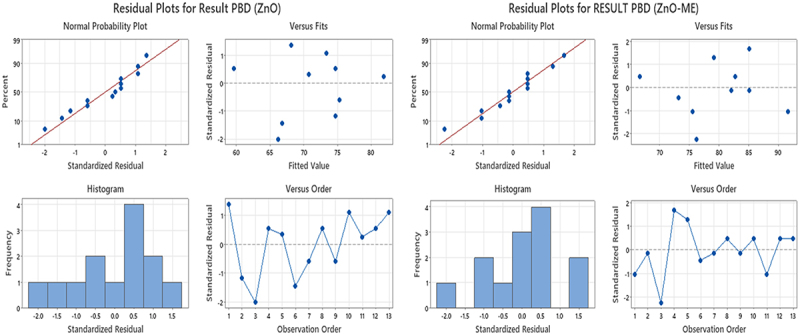
Residual plots for PBD for ZnO and ZnO-ME nanoparticles.

The high *F*-value [315.97 for ZnO and 461.12 for ZnO-ME] and probability *p*-value <0.0001 of the model, as determined by the analysis of the experimental data for Sulfur Black dye adsorption, make it clear that the model is highly significant. A non-significant lack of fit test was validated with a low *F*-value of >0.0500. The fact that the ‘adjusted *R*^2^’ is quite close to the ‘predicted R^2^’ indicates that the experimental data fits the model very well. The removal % data are perfectly agreed with the model’s anticipated value, as indicated by *R*^2^ value. The linear correlation was depicted by the normal probability plot, showing that the actual experimental values of sulfur black dye adsorption are in good agreement with those predicted by the model, as shown in Figure 6. The results of the PB design can be seen graphically using Pareto charts of standardized effects and normal plot of standardized effects and main effects. Based on standardized effect Pareto plots, the variables that are above the red reference line are statistically significant [42]. The length of the depicted bar represents the parameter’s weighting as illustrated in Figure S5 (Supplementary data). The following factors were impacted on the sulfur black dye’s adsorption rate: In ZnO-ME nanoparticles, the initial dye conc. (B) impact was found higher on the dye adsorption rate which was determined by the 95% confidence interval followed by contact time (C) and dose (A), respectively. For ZnO-ME nanoparticles, the sulfur black dye adsorption rate followed the following order: C (Contact time) > A (dosage) > B (initial dye conc.). At the 95% confidence level, the effect of contact time on the dye adsorption rate was shown to be most significant.

The direction of the effect is additionally shown by the normal plot of the standardized effects (Figure S6). The standardized effects of factors A, B, and C were positive for both nanoparticles. The response increases when these shifts take place from the component’s low level to its high level. The parameters’ impact between high and low settings is depicted in the main impact plot (Figure S7). The blue circles (Corner point type) on either side of the line represent the mean of the values of the high and low sets. These two points are connected by the blue line. A line with a high slope denotes a considerable influence of the parameter on the response.

#### 3.3.3. Full factorial design (FFD)

Based on the results of a three-factor experiment, a two-level FFD factorial design with nine runs was used to unbiased screen the variables that significantly influence the sulfur black dye adsorption onto ZnO and ZnO-ME nanoparticles. To prevent systemic errors, the experimentation was done in a randomized order. Factorial plots, including the Pareto chart, normal probability, main effect, and surface plots, factors that affect the amount of dye that is adsorbed onto nanoparticles were assessed. The significance of the influence on the adsorption rate was examined using ANOVA and *p*-value significant levels (refer to Table 1). Additionally, the correlation coefficient (*R*^2^) of the model for ZnO-ME and ZnO nanoparticles was 0.994 and 0.999, respectively, confirming the well-fitted statistical model. Also, the predicted *R*^2^ value is close to the adjusted *R*^2^ (adj). Equation 14 and Equation 15) are used to represent the dye adsorption by both nanoparticles:

(14) $$\mathrm{Removal}\;\;\mathrm{efficiency}\;\left(\mathrm{ZnO}-\mathrm{ME}\right)\;=\;59.535+0.3375\;\mathrm{Dose}\left(\mathrm{mg}\right)\;+0.10278\;\mathrm{Conc}.\left(\mathrm{mg}/L\right)\;+0.07292\;\mathrm{Contact}\;\mathrm{time}\left(\min\right)$$

(15) $$\mathrm{Removal}\;\;\mathrm{efficiency}\;\left(\mathrm{ZnO}-\mathrm{ME}\right)\;=\;53.806+0.2861\;\mathrm{Dose}\left(\mathrm{mg}\right)+0.04861\;\mathrm{Conc}.\left(\mathrm{mg}/L\right)\;+0.07292\;\mathrm{Contact}\;\mathrm{time}\left(\min\right)+0.001389\;\mathrm{Dose}\left(\mathrm{mg}\right)*\mathrm{Conc}.\left(\mathrm{mg}/L\right)$$

Figure S8 illustrates the main effects of each parameter on dye adsorption, while the regression analysis findings are visualized through main effect plots. Only the significant factors are revealed at the 95% confidence level. The ANOVA was also used to create the main effect charts. The relative importance of the primary impacts and their interactions might be seen on the Pareto chart (Figure S9). These values are shown in the Pareto graphic’s horizontal columns for each effect. A Student’s *t*-test was used to see whether the calculated effects were statistically different from zero. The significant values are seen above a reference line and inside the 95% confidence interval, as shown in Figure S9 [43]. The three main components (A, B, and C) and their interactions (AB only for ZnO-ME) were significant at the level of 0.05.

**Figure 7.**
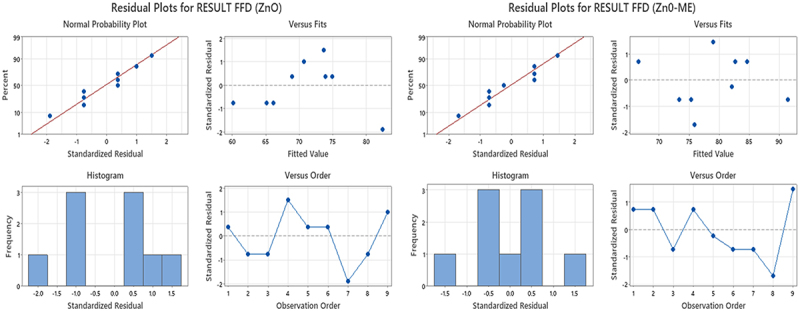
Residual plots for FFD for ZnO and ZnO-ME nanoparticles.

It is uncertain whether these results are ‘real’ or ‘chance.’ The ‘real’ impacts are identified using a normal probability plot in Figure 7. Each effect has a single point on the plot. In accordance with the normal probability plots, the estimated factors that do not show a substantial impact on the response variables are represented by the points that lie along a line that passes through the center of the middle group of points. According to Geyikci and Büyükgüngör (2013), points that are far from the line most likely indicate the ‘real’ effects of factor [44]. The normal probability plot of residuals for the adsorption rate demonstrated that the set of experimental results closely matched the expected distribution. A normal distribution is suggested by the fact that the majority of the experimental points are reasonably aligned.

Surface plots of the response functions show the major impacts of the variables as well as their interactions. The response surface plots depicting the adsorption rate are presented in (Figure S10). The standardized effects plot’s normal plot further displays the effect’s direction (Figure S11). Factors A, B, and C (AB only in the case of ZnO) have favorable standardized impacts for both nanoparticles. When these changes from the low level to the high level of the component take place, the reaction increases.

### 3.4. Statistical comparison, performance of optimising models and comparison with other studies

In addition to analyzing the fit of each model using ANOVA, the models’ propensities for forecasting the adsorption of sulfur black dye from the aqueous matrix were statistically assessed using the coefficient of determination (*R*^2^) and standard deviation (SD). Based on the R^2^ and SD values, the accuracy of each model was compared and summarized in Table 2. The FFD model clearly outperformed the other model for data fitting based on the *R*^2^ value for ZnO (0.998) and ZnO-ME (0.993) nanoparticles, which means FFD well predicted the outcomes. All other models also offered good-quality predictions (*R*^2^) for the parameters within the design range. In conclusion, FFD also performed well compared to other models while simulating the adsorption process. Interestingly, all the models show the same optimized conditions and removal efficiency on these optimized conditions (i.e. 71% for ZnO and 80% for ZnO-ME). These results are comparable with other studies using zinc oxide nanoparticles for dye adsorption, as given in Table 3.

**Table 3.** Comparison of adsorption percentage of zinc oxide nanoparticles for various dyes.

Nanomaterials	Dye	Adsorption (%)	Reference
ZnO	Ismate violet 2R (IV2R) dye (aqueous solution)	91.5	[45]
ZnO	Textile industry effluent (real)	65.6	[45]
Lemon peel beads-doped zinc oxide (LBZ) NPs	Reactive Blue 4 (RB4) dye	66.64	[46]
ZnO	Congo Red dye	87.3	[47]
ZnO-loaded zeolite	Crystal violet	69.7	[48]
ZnO	Sulfur black	71	Present study
ZnO-ME	Sulfur black	80	Present study

### 3.5. Adsorption isotherm model

Three isotherm models – Langmuir, Freundlich, and Temkin – were used to identify how the adsorption mechanism. These isotherm models were applied to the experimental datasets generated using FFD. Equation 16, Equation 17, and Equation 18 offer the linear forms of the models under study.

(16) $$\frac{C_{e}}{q_{e}}=\frac{1}{q_{\max}b}+\frac{C_{e}}{q_{\max}}$$

(17) $$\log q_{e}=\log K_{F}+\frac{1}{n}\log C_{e}$$

(18) $$q_{e}=\mathrm{Bln}K_{T}+\mathrm{Bln}C_{e}$$

Where *C*_*e*_ (mg/L) is the amount of remaining adsorbate in solution and *q*_*e*_ (mg/g) denotes the number of dye molecules that interact with the adsorbent. The maximum monolayer adsorption capacity is denoted by the symbol *q*_max_ (mg/g), and the Langmuir constant *b* (J/mol) is related to the affinity of binding sites. *B = RT*/*b*, where *b* is the equilibrium binding constant, *R* is the gas constant, and *K*_*T*_ (L/mg) is the Temkin constant related to the heat of adsorption. *T* (K) is the absolute temperature and *Kf* (mg/g (L/mg)1/^*n*^) is the Freundlich constant.

In order to determine the values of these parameters and correlation coefficients (*R*^2^), linear equations were generated using the corresponding plots of *C*_*e*_*/q*_*e*_ versus *C*_*e*_, log *q*_*e*_ versus log *C*_*e*_, and *q*_*e*_ versus ln*C*_*e*_ (Figure S12 and Figure S13). With the help of the regression coefficient and parameter values, the models’ appropriateness is demonstrated. After comparison, it was shown that the Freundlich model better explained the behavior of dye adsorption, demonstrating multilayer sulfur black dye adsorption onto the ZnO and ZnO-ME nanoparticles. Its coefficient of determination was greater than other examined models for ZnO and ZnO-ME nanoparticles, respectively, at 0.9625 and 0.9549 as can be seen in Table 4. Furthermore, multilayer adsorption is thought to occur on heterogeneous surfaces and on the surface sites of the adsorbent that have different binding energies, according to the Freundlich isotherm, an empirical isotherm model. Additionally, it states that when the concentration of dye in an aqueous solution rises, adsorption increases indefinitely. A heterogeneous adsorption process, which is favored by the Freundlich isotherm, is assumed to occur when there is an uneven distribution of heat on the adsorbent’s surface. More variability in the adsorbent surface is indicated by a slope value that is closer to 0 [49].

**Table 4.** Isotherm parameters for sulfur black dye adsorption on ZnO and ZnO-ME.

		Parameter value	
Isotherm Model	Parameter	ZnO	ZnO-ME
Langmuir	*q*_max_ (mg/g)	2.774	4.123
	*R*^2^	0.324	0.267
Freundlich	*n*	0.858	0.767
	*Kf* (mg/g)	2.926	3.88
	*R*^2^	0.9625	0.9549
Temkin	*b* (J/mol)	74.52	95.442
	*Kt* (L/mg)	0.339	0.432
	*R*^2^	0.670	0.679

### 3.6. Adsorption mechanism

**Figure 8.**
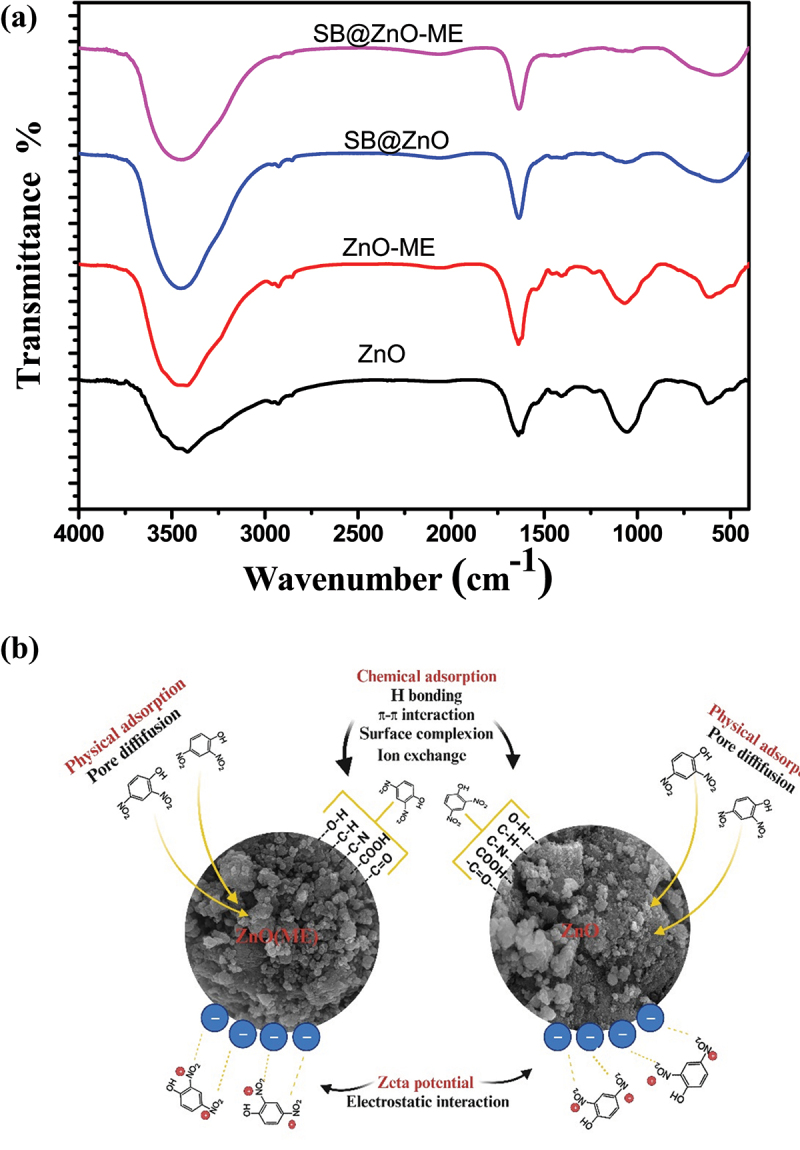
(a) FT-IR spectra of nanomaterials before and after adsorption; (b) potential adsorption mechanism.

Analysis of the dye adsorption mechanism on adsorbent surfaces is challenging because of the complex interactions between the adsorbent and adsorbate. The features of the adsorbent surface, functional groups, and molecular structure of the dye molecules all play a key part in the interaction and binding of the dye molecules to the surface of the adsorbent. The hypothesized method may be demonstrated by comparing the FTIR spectra of ZnO and ZnO-ME nanoparticles before and after dye adsorption, as shown in Figure 8a. The IR peak of both nanoparticles prior to adsorption has already been covered in Section 3.2.3. The hydrogen bond interaction between the dye mlecules and adsorbent surface may be the cause of the rise in band strength at 3440 cm^−1^ in the FTIR spectra of sulfur black dye adsorbed ZnO and ZnO-ME nanoparticles. Kataria et al., (2022) have demonstrated the significance of the O–H group in the adsorption of dye molecules on zinc oxide nanoparticles [3]. This study illustrates its function in the adsorption of color molecules as ZnO-ME exhibits more dye removal than ZnO due to a sharp increase in peaks at 3440 cm^−1^. Strong peaks at 2926.26 cm^−1^ and 2336 cm^−1^ are associated to the –C=C stretching vibration of alkynes, and a minor decline in these peaks was also noticed, demonstrating the function of –C=C stretching in adsorption. The C–H bond is responsible for the peak between 1050 and 900 cm^−1^, and completely diminishing of these peaks highlights its function in the adsorption of the sulfur black dye due to hydrogen bonding. Peak at 1633.39 cm^−1^ represents amine group and alteration in intensity and slight shift in position after dye adsorption shows its role in adsorption process and formation of amide group [50]. π–π interaction is presented between the benzene rings of the dye and the adsorbent. A sharp broad peak at 619–480 cm^−1^ corresponds to the Zn–O stretching, FTIR of dye-laden ZnO and ZnO-ME NPs showed slight change in the peak, indicating the significance of it in the process of dye adsorption. Adsorption of dye molecules onto ZnO and ZnO-ME NPs showed hydrogen bonding, electrostatic attraction, π–π interaction in the adsorption of sulfur black dye which suggest chemisorption [51].

Zeta potential values show negative charge on the surface of nanomaterials and dye molecules show positive charge (N^+^) on their surface, showing electrostatic attraction and this helps in the adsorption of dye [52]. From the freundlich adsorption isotherm, it can be seen the *K_f_* value for ZnO and ZnO-ME NPs 2.9 and 3.8, respectively, which shows the physisorption through weak Van der Waals forces. Further, the role of physisorption is shown by the small size of the NPs which is shown by the blue shift of UV–VIS and the small size of NPs can also be seen in the SEM images. BET surface area of 7.617 and 33.635 m^2^/g for ZnO and ZnO-ME, respectively, also supports physisorption due to high surface area. A potential mechanism for color adsorption for both the nanocomposites is depicted in Figure 8b.

## 4. Conclusion

ZnO and ZnO-ME nanoparticles having diameters of 22.33 and 39 nm, respectively, with mesoporous structure were synthesized using *Bacillus paramycoides* for SB dye removal. Out of used optimized models (CCD, PBD, and FFD), FFD showed better predictability, with R^2^ values of 0.9985 and 0.9937 for ZnO and ZnO-ME, respectively, and removal efficiency on these optimized conditions (i.e. 71% for ZnO and 80% for ZnO-ME). The Freundlich isotherm model accurately predicted the physisorption of sulfur black dye, with *R*^2^ values of 0.96 for ZnO and 0.95 for ZnO-ME, and maximum adsorption capacities of 2.77 mg/g for ZnO and 4.12 mg/g for ZnO-ME.

## Supplementary Material

Supplementary Information-clean.docx

## Data Availability

The authors confirm that the data supporting the findings of this study are available within the article and its supplementary materials.

## References

[1] Ohale PE, Onu CE, Nwabanne JT, et al. A comparative optimization and modeling of ammonia–nitrogen adsorption from abattoir wastewater using a novel iron-functionalized crab shell. Appl Water Sci. 2022;12(8):193. doi: 10.1007/s13201-022-01713-4

[2] Yadav S, Punia S, Sharma HR, et al. Nano-remediation for the decolourisation of textile effluents: a review. Nanofabrication. 2022;7:217–20. doi: 10.37819/nanofab.007.226

[3] Kataria N, Chauhan AK, Garg VK, et al. Sequestration of heavy metals from contaminated water using magnetic carbon nanocomposites. J Hazard Mater Adv. 2022;6:100066. doi: 10.1016/j.hazadv.2022.100066

[4] Baştürk E, Alver A. Modeling azo dye removal by sono-Fenton processes using response surface methodology and artificial neural network approaches. J Environ Manage. 2019;248:109300. doi: 10.1016/j.jenvman.2019.109300 31351408

[5] Darwish AAA, Rashad M, Al-Aoh HA. Methyl orange adsorption comparison on nanoparticles: isotherm, kinetics, and thermodynamic studies. Dyes Pigm. 2019;160:563–71. doi: 10.1016/j.dyepig.2018.08.045

[6] Chauhan, AK , Kataria N, Gupta, R, Garg VK, et al. Biogenic fabrication of ZnO@EC and MgO@EC using Eucalyptus leaf extract for the removal of hexavalent chromium Cr(VI) ions from water. Environmental Science and Pollution Research. 2023;30:124884–124901. doi: 10.1007/s11356-022-24967-6 36596976

[7] Li W, Mu B, Yang Y. Feasibility of industrial-scale treatment of dye wastewater via bio-adsorption technology. Bioresour Technol. 2019;277:157–70. doi: 10.1016/j.biortech.2019.01.002 30638884

[8] Huang Y, Zeng H, Xie L, et al. Super-assembled chiral mesostructured heteromembranes for smart and sensitive couple-accelerated enantioseparation. J Am Chem Soc. 2022;144(30):13794–13805. doi: 10.1021/jacs.2c04862 35830296

[9] Kim D-Y, Sharma SK, Rasool K, et al. Development of novel peptide-modified silver nanoparticle-based rapid biosensors for detecting aminoglycoside antibiotics. J Agric Food Chem. 2023;71(34):12883–12898. doi: 10.1021/acs.jafc.3c03565 37603424

[10] Zahmatkesh S, Hajiaghaei-Keshteli M, Bokhari A, Sundaramurthy S, Panneerselvam B, Rezakhani Y. Wastewater treatment with nanomaterials for the future: a state-of-the-art review. Environ Res. 2023;216:114652. doi: 10.1016/j.envres.2022.114652 36309214

[11] Tran TV, Nguyen DTC, Kumar PS, et al. Green synthesis of ZrO2 nanoparticles and nanocomposites for biomedical and environmental applications: a review. Environ Chem Lett. 2022;20(2):1309–1331. doi: 10.1007/s10311-021-01367-9 35035338 PMC8741578

[12] Pugazhendhi A, Prabakar D, Jacob JM, Karuppusamy I, Saratale RG. Synthesis and characterization of silver nanoparticles using Gelidium amansii and its antimicrobial property against various pathogenic bacteria. Microb Pathog. 2018;114:41–5. doi: 10.1016/j.micpath.2017.11.013 29146498

[13] Hammad EN, Salem SS, Zohair MM, et al. Purpureocillium lilacinum mediated biosynthesis copper oxide nanoparticles with promising removal of dyes. Biointerface Res Appl Chem. 2022;12:1397–1404. doi: 10.33263/BRIAC122.13971404

[14] Weisburg WG, Barns SM, Pelletier DA, et al. 16S ribosomal DNA amplification for phylogenetic study. J Bacteriol. 1991;173(2):697–703. doi: 10.1128/jb.173.2.697-703.1991 1987160 PMC207061

[15] Kailasa SK, Borse S, Koduru JR, et al. Biomolecules as promising ligands in the synthesis of metal nanoclusters: sensing, bioimaging and catalytic applications. Trends Environ Anal Chem. 2021;32:e00140. doi: 10.1016/j.teac.2021.e00140

[16] Zeng H, Zhou S, Xie L, Liang Q, Zhang X, Yan M, et al. Super-assembled mesoporous thin films with asymmetric nanofluidic channels for sensitive and reversible electrical sensing. Biosens Bioelectron. 2023;222:114985. doi: 10.1016/j.bios.2022.114985 36493724

[17] Lim S, Kim JH, Park H, et al. Role of electrostatic interactions in the adsorption of dye molecules by Ti3C2-MXenes. RSC Adv. 2021;11(11):6201–6211. doi: 10.1039/D0RA10876F 35423145 PMC8694804

[18] Chollom MN, Rathilal S, Swalaha FM, et al. Comparison of response surface methods for the optimization of an upflow anaerobic sludge blanket for the treatment of slaughterhouse wastewater. Environ Eng Res. 2020;25(1):114–122. doi: 10.4491/eer.2018.366

[19] Boateng ID, Yang X-M. Process optimization of intermediate-wave infrared drying: screening by plackett–Burman; comparison of Box–Behnken and central composite design and evaluation: a case study. Ind Crops Prod. 2021;162:113287. doi: 10.1016/j.indcrop.2021.113287

[20] Ali A, Ing AWC, Abdullah WRW, et al. Preparation of high-performance adsorbent from low-cost agricultural waste (peanut husk) using full factorial design: application to dye removal. Biointerface Res Appl Chem. 2020;10:6619–6628. doi: 10.33263/BRIAC106.66196628

[21] Ghosh A, Das P, Sinha K. Modeling of biosorption of Cu(II) by alkali-modified spent tea leaves using response surface methodology (RSM) and artificial neural network (ANN). Appl Water Sci. 2015;5(2):191–9. doi: 10.1007/s13201-014-0180-z

[22] Weber TW, Chakravorti RK. Pore and solid diffusion models for fixed-bed adsorbers. AichE J. 1974;20(2):228–238. doi: 10.1002/aic.690200204

[23] Ali K, De P, Bhowmik GC, et al. Adsorption behavior of methylene blue onto gellan gum-bentonite composite beads for bioremediation application. World J Pharm Sci. 2016:68–72.

[24] Tempkin M, Pyzhev V. Kinetics of ammonia synthesis on promoted iron catalyst. J Acta Phys Chim USSR. 1940;12:327.

[25] Bhattacharjee S. DLS and zeta potential – what they are and what they are not? J Control Release. 2016;235:337–51. doi: 10.1016/j.jconrel.2016.06.017 27297779

[26] Ahmad A, Wei Y, Syed F, Tahir K, Rehman AU, Khan A, et al. The effects of bacteria-nanoparticles interface on the antibacterial activity of green synthesized silver nanoparticles. Microb Pathog. 2017;102:133–42. doi: 10.1016/j.micpath.2016.11.030 27916692

[27] Senthilkumar N, Nandhakumar E, Priya P, et al. Synthesis of ZnO nanoparticles using leaf extract of Tectona grandis (L.) and their anti-bacterial, anti-arthritic, anti-oxidant and in vitro cytotoxicity activities. New J Chem. 2017;41(18):10347–10356. doi: 10.1039/C7NJ02664A

[28] Shanavas S, Duraimurugan J, Kumar GS, et al. Ecofriendly green synthesis of ZnO nanostructures using Artabotrys hexapetalu and Bambusa vulgaris plant extract and investigation on their photocatalytic and antibacterial activity. Mater Res Express. 2019;6(10):105098. doi: 10.1088/2053-1591/ab3efe

[29] Ahmad W, Kalra D. Green synthesis, characterization and anti microbial activities of ZnO nanoparticles using Euphorbia hirta leaf extract. J King Saud Univ Sci. 2020;32(4):2358–2364. doi: 10.1016/j.jksus.2020.03.014

[30] Venu Gopal VR, Kamila S. Effect of temperature on the morphology of ZnO nanoparticles: a comparative study. Appl Nanosci. 2017;7(3–4):75–82. doi: 10.1007/s13204-017-0553-3

[31] Rajabairavi N, Raju CS, Karthikeyan C, et al. Biosynthesis of novel zinc oxide nanoparticles (ZnO NPs) using endophytic bacteria Sphingobacterium thalpophilum. Cham: Springer International Publishing; 2017. p. 245–254.

[32] Siddique A, Nayak AK, Singh J. Synthesis of FeCl_3_-activated carbon derived from waste citrus limetta peels for removal of fluoride: an eco-friendly approach for the treatment of groundwater and bio-waste collectively. Groundwater Sust Develop. 2020;10:100339. doi: 10.1016/j.gsd.2020.100339

[33] Vijayakumar S, Mahadevan S, Arulmozhi P, Sriram S, Praseetha PK. Green synthesis of zinc oxide nanoparticles using Atalantia monophylla leaf extracts: characterization and antimicrobial analysis. Mater Sci Semicond Process. 2018;82:39–45. doi: 10.1016/j.mssp.2018.03.017

[34] Vaseem M, Lee K-M, Shin J-K, et al. Synthesis of ZnO nanoparticles and their ink-jetting behavior. J Nanosci Nanotechnol. 2012;12(3):2380–2386. doi: 10.1166/jnn.2012.5693 22755062

[35] Gan Y, Gu F, Han D, et al. Biomimetic synthesis of zinc oxide 3D architectures with gelatin as matrix. J Nanomater. 2010;2010:1–7. Article 7. doi: 10.1155/2010/289173

[36] Meraat R, Issazadeh K, Abdolahzadeh Ziabari A, et al. Rapid detection of Escherichia coli by β-galactosidase biosensor based on ZnO NPs and MWCNTs: a comparative study. Curr Microbiol. 2020;77(10):2633–2641. doi: 10.1007/s00284-020-02040-0 32444907

[37] Singh AV, Patil R, Anand A, et al. Biological synthesis of copper oxide nano particles using Escherichia coli. Curr Nanosci. 2010;6(4):365–369. doi: 10.2174/157341310791659062

[38] Shantkriti S, Rani P. Biological synthesis of copper nanoparticles using Pseudomonas fluorescens. Int J Curr Microbiol App Sci. 2014;3:374–383.

[39] Varadavenkatesan T, Lyubchik E, Pai S, Pugazhendhi A, Vinayagam R, Selvaraj R. Photocatalytic degradation of rhodamine B by zinc oxide nanoparticles synthesized using the leaf extract of Cyanometra ramiflora. J Photochem Photobiol B Biol. 2019;199:111621. doi: 10.1016/j.jphotobiol.2019.111621 31610434

[40] Pai S, Kini SM, Narasimhan MK, Pugazhendhi A, Selvaraj R. Structural characterization and adsorptive ability of green synthesized Fe_3_O_4_ nanoparticles to remove acid blue 113 dye. Surf Interfaces. 2021;23:100947. doi: 10.1016/j.surfin.2021.100947

[41] Lingamdinne LP, Vemula KR, Chang Y-Y, et al. Process optimization and modeling of lead removal using iron oxide nanocomposites generated from bio-waste mass. Chemosphere. 2020;243:125257. doi: 10.1016/j.chemosphere.2019.125257 31726263

[42] Haddar W, Baaka N, Meksi N, Elksibi I, Farouk Mhenni M. Optimization of an ecofriendly dyeing process using the wastewater of the olive oil industry as natural dyes for acrylic fibres. J Clean Prod. 2014;66:546–54. doi: 10.1016/j.jclepro.2013.11.017

[43] Bingol D, Tekin N, Alkan M. Brilliant yellow dye adsorption onto sepiolite using a full factorial design. Appl Clay Sci. 2010;50(3):315–321. doi: 10.1016/j.clay.2010.08.015

[44] Geyikci F, Büyükgüngör H. Factorial experimental design for adsorption silver ions from water onto montmorillonite. Acta Geodyn Geomater. 2013;10:363–370. doi: 10.13168/AGG.2013.0035

[45] Al-Arjan WS. Zinc oxide nanoparticles and their application in adsorption of toxic dye from aqueous solution. MDPI Polymer. 2022;14(15):3086. doi: 10.3390/polym14153086 PMC9370170 35956598

[46] Praipipat P, Ngamsurach P, Prasongdee V. Comparative reactive blue 4 dye removal by lemon peel bead doping with iron(III) oxide-hydroxide and zinc oxide. ACS Omega. 2022;7(45):41744–41758. doi: 10.1021/acsomega.2c05956 36406531 PMC9670269

[47] Chauhan AK, Kataria N, Garg VK. Green fabrication of ZnO nanoparticles using Eucalyptus spp. leaves extract and their application in wastewater remediation. Chemosphere. 2020;247:125803. doi: 10.1016/j.chemosphere.2019.125803 31972482

[48] Elfeky AS, Youssef HF, Elzaref AS. Adsorption of dye from wastewater onto ZnO nanoparticles-loaded zeolite: kinetic, thermodynamic and isotherm studies. Zeitschrift für Physikalische Chemie. 2020;234(2):255–278. doi: 10.1515/zpch-2018-1342

[49] Rawat S, Samreen K, Nayak AK, et al. Fabrication of iron nanoparticles using parthenium: a combinatorial eco-innovative approach to eradicate crystal violet dye and phosphate from the aqueous environment. Environ Nanotechnol Monit Manage. 2021;15:100426. doi: 10.1016/j.enmm.2021.100426

[50] Prasannamedha G, Kumar PS, Mehala R, Sharumitha TJ, Surendhar D. Enhanced adsorptive removal of sulfamethoxazole from water using biochar derived from hydrothermal carbonization of sugarcane bagasse. J Hazard Mater. 2021;407:124825. doi: 10.1016/j.jhazmat.2020.124825 33359976

[51] Li X, Xie L, Yang X, et al. Adsorption behavior and mechanism of β-cyclodextrin–styrene-based polymer for cationic dyes. RSC Adv. 2018;8(70):40321–40329. doi: 10.1039/C8RA07709F 35558233 PMC9091483

[52] Pinheiro D, Sunaja Devi KR, Jose A, et al. Effect of surface charge and other critical parameters on the adsorption of dyes on SLS coated ZnO nanoparticles and optimization using response surface methodology. J Environ Chem Eng. 2020;8(4):103987. doi: 10.1016/j.jece.2020.103987

