# Supplementary material for: Biogenic engineered zinc oxide nanoparticle for sulfur black dye removal from contaminated wastewater: comparative optimization, simulation modeling, and isotherms
Source: Bioengineered. 2024 Mar 11;15(1):2325721. doi: 10.1080/21655979.2024.2325721 (PMC10936635; doi:10.1080/21655979.2024.2325721)
Supplement: Supplementary Information-clean.docx [file KBIE_A_2325721_SM3595.docx]

**Biogenic engineered zine oxide nano****particle for sulfur black dye removal from contaminated wastewater: comparative optimisation, simulation modelling and isotherms**

**Supplementary Data**

Sangita Yadav ^a^, Subhash Chander ^a^, Asha Gupta ^a, *^, Navish Kataria ^b, *^, Kuan Shiong Khoo ^c, d, *^

^a^ Department of Environmental Science and Engineering, Guru Jambheswar University of Science & Technology, Hisar-125001, Haryana, India

^b^ Department of Environmental Science and Engineering, J. C. Bose University of Science and Technology, YMCA, Faridabad-121006, Haryana, India

^c^ Department of Chemical Engineering and Materials Science, Yuan Ze University, Taoyuan, Taiwan

^d^ Centre for Herbal Pharmacology and Environmental Sustainability, Chettinad Hospital and Research Institute, Chettinad Academy of Research and Education, Kelambakkam-603103, Tamil Nadu, India

***Corresponding authors:**

1. **Prof. Dr. Asha Gupta****,** Email: [guptaasha.env@gmail.com](mailto:guptaasha.env@gmail.com)
2. **Assistant Professor Dr. Navish Kataria,** Email: [navishkataria@jcboseust.ac.in](mailto:navishkataria@jcboseust.ac.in)
3. **Assistant Professor ChM. Dr. Kuan Shiong Khoo,** Tel: +886-3-4638800 Email: [kuanshiong.khoo@saturn.yzu.edu.tw](mailto:kuanshiong.khoo@saturn.yzu.edu.tw) or [kuanshiong.khoo@hotmail.com](mailto:kuanshiong.khoo@hotmail.com)


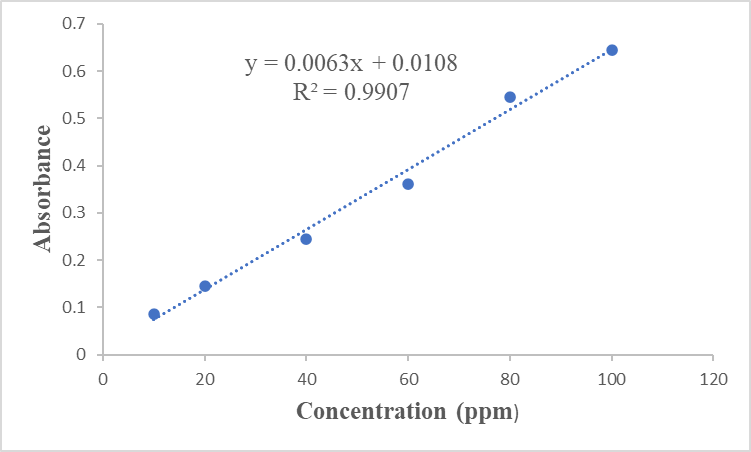


**Fig. S1** Standard curve for sulfur black dye


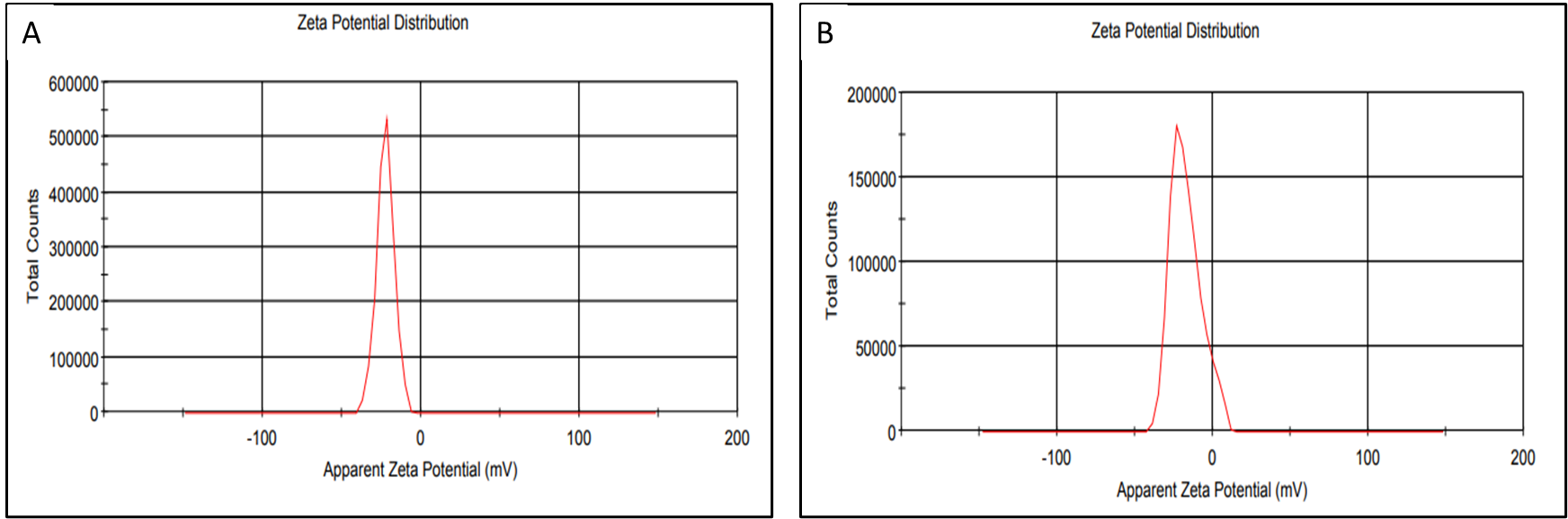


**Fig.S2** Zeta potential value via dynamic light scattering A) ZnO and B) ZnO(ME)

**
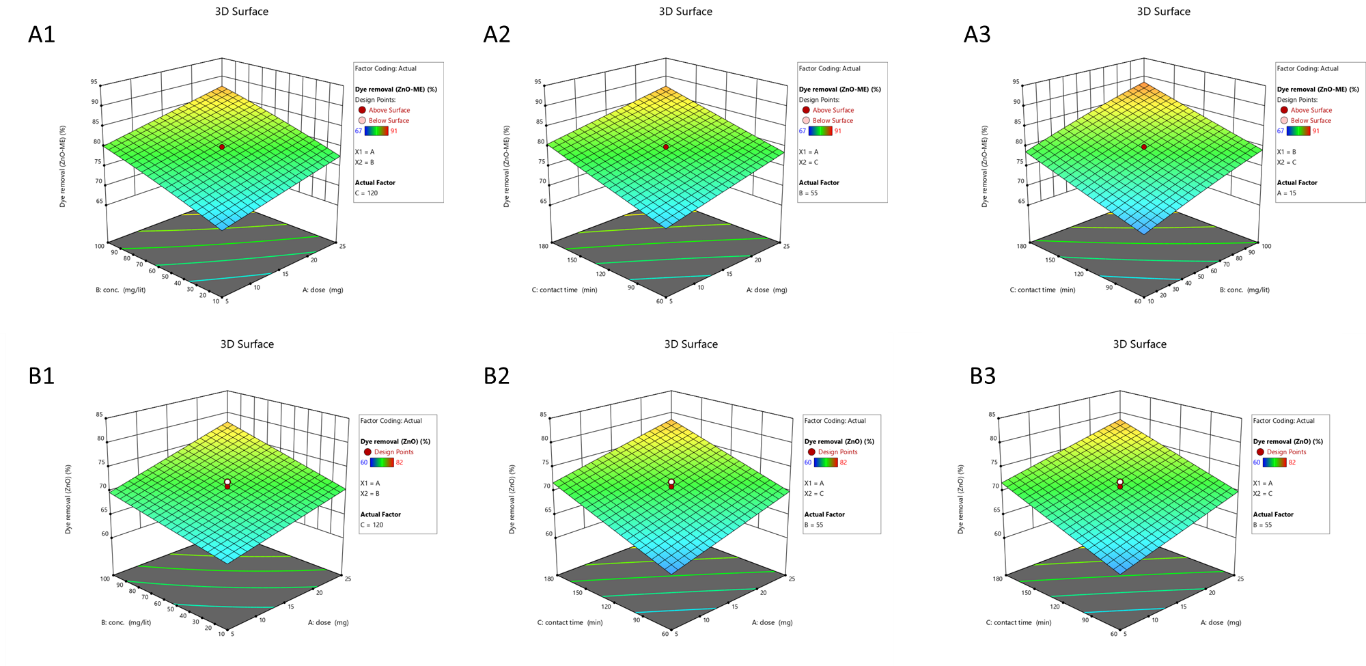
**

**Fig.S3** CCD-Three-dimensional response surfaces for sulfur black dye adsorption using (A) ZnO-ME: (A1) 3-D plot between dose, initial dye conc. and dye removal; (A2) 3-D plot between dose, contact time and dye removal; (A3) 3-D plot between initial dye conc., contact time and dye removal; and (B) ZnO nanoparticle: (B1) 3-D plot between dose, initial dye conc. and dye removal; (B2) 3-D plot between dose, contact time and dye removal; (B3) 3-D plot between initial dye conc., contact time and dye removal


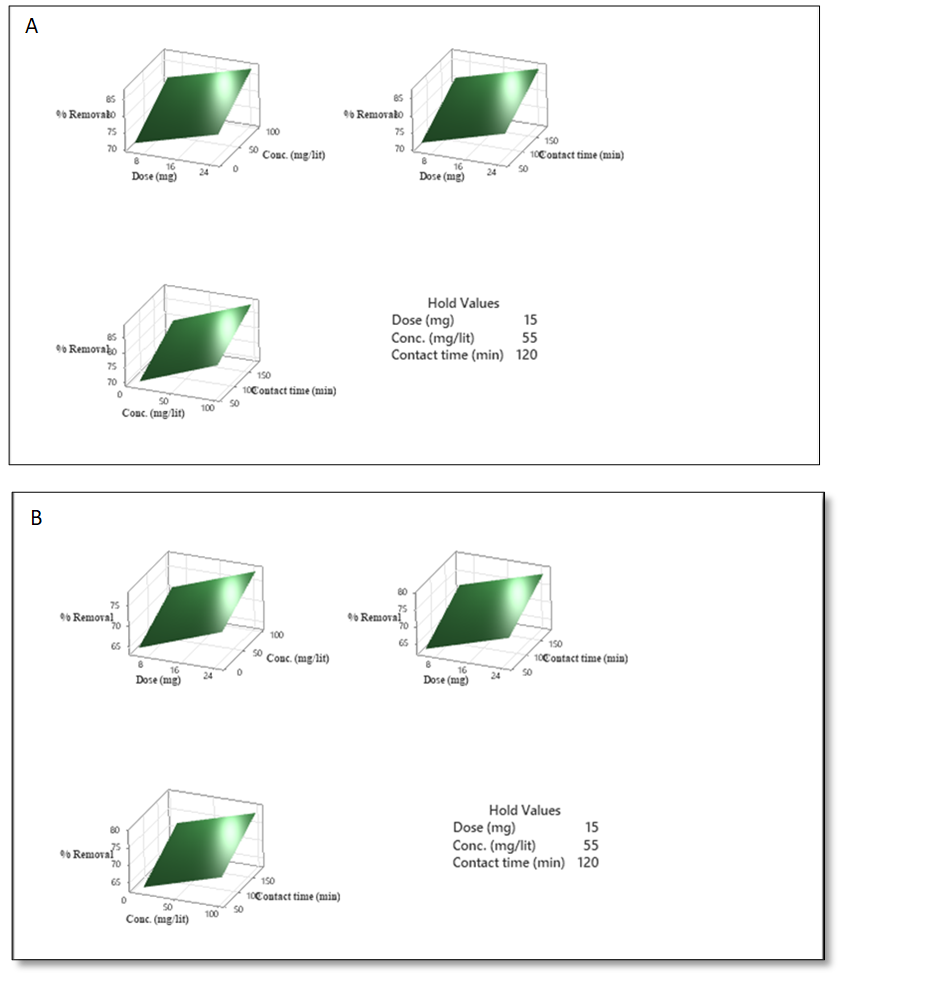


**Fig.S4** PBD-Three-dimensional response surfaces for sulfur black dye adsorption between three parameters, i.e., dose, initial dye conc. and contact time at hold values and percentage dye removal using (A) ZnO(ME) and (B) ZnO nanomaterials


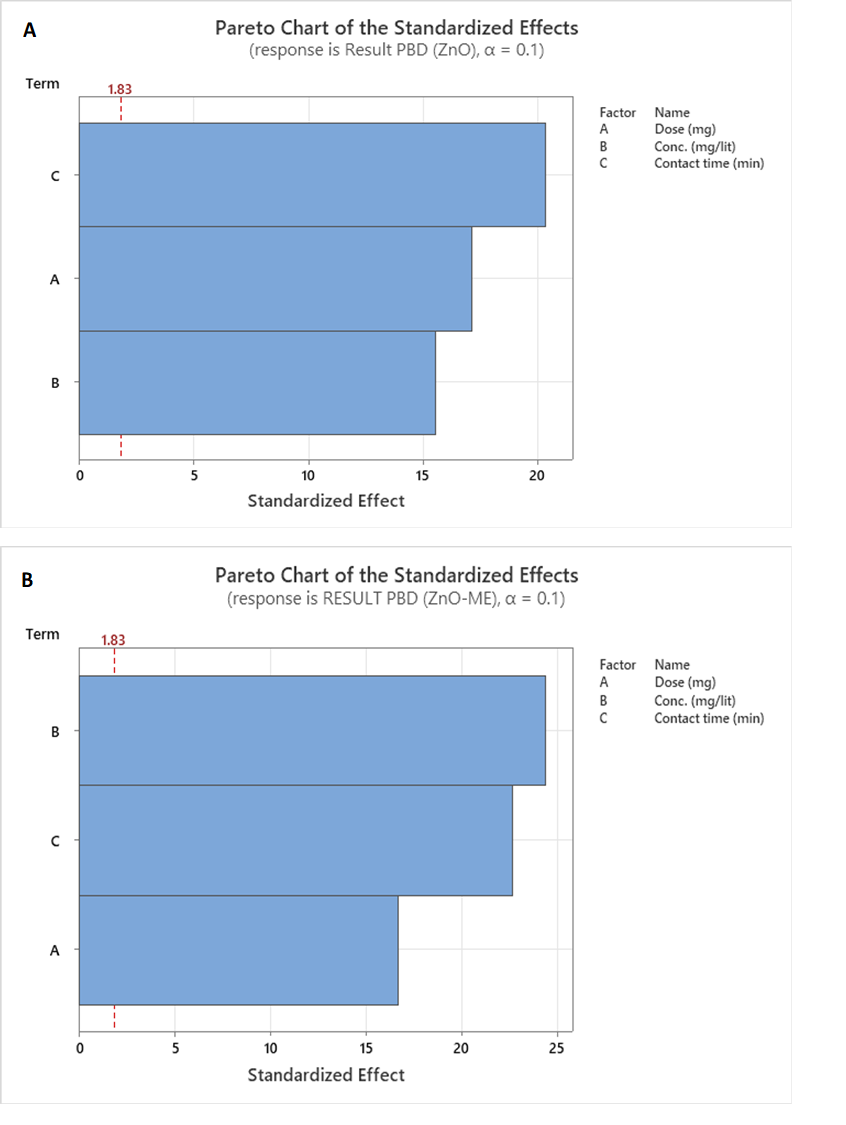


**Fig.S5** Pareto plots of (A) ZnO and (B) ZnO-ME nanoparticles between the parameters adsorbent dose, initial dye conc. and contact on the Y-axis and the standardized effect on the X-axis to show the effect of each parameter on the dye adsorption.


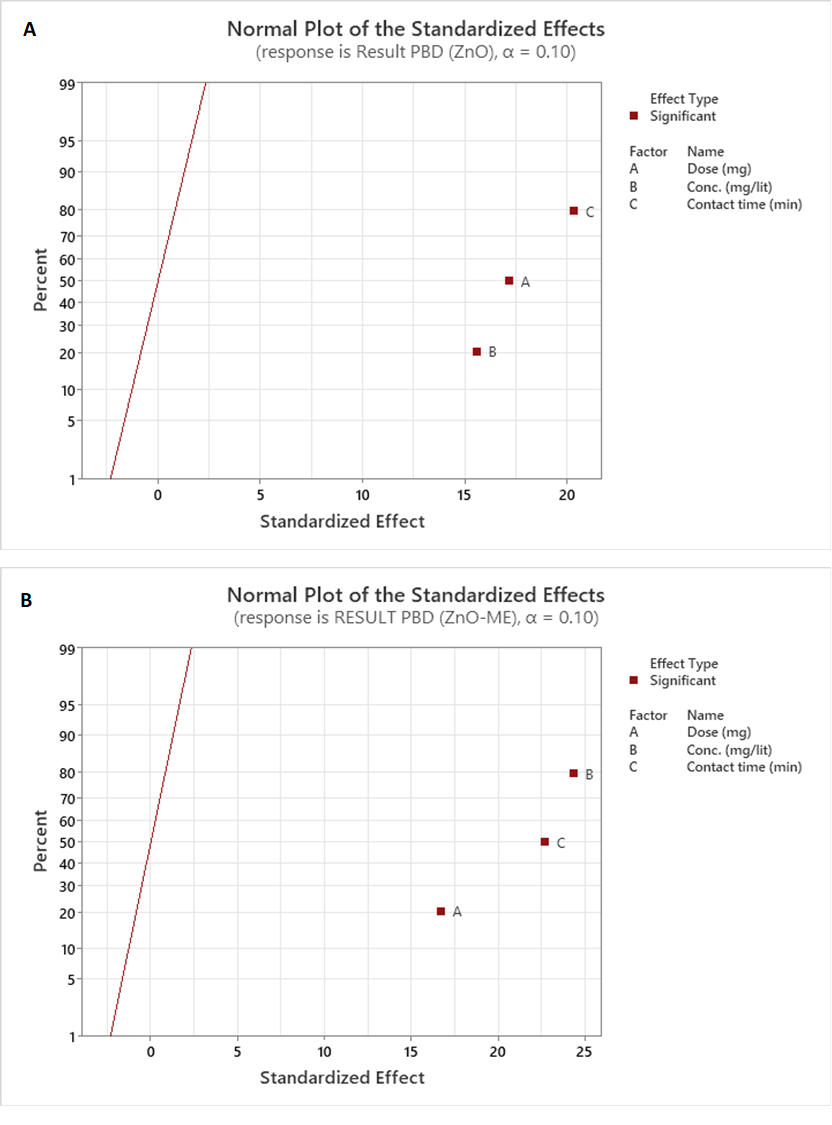


**Fig.S6** Normal plots of the standardised effects between the standardized effect on the X-axis and percentage effect on Y-axis for the experimental parameters, i.e., dose (A), initial dye conc. (B) and contact time (C) : (A) ZnO and (B) ZnO-ME nanoparticles


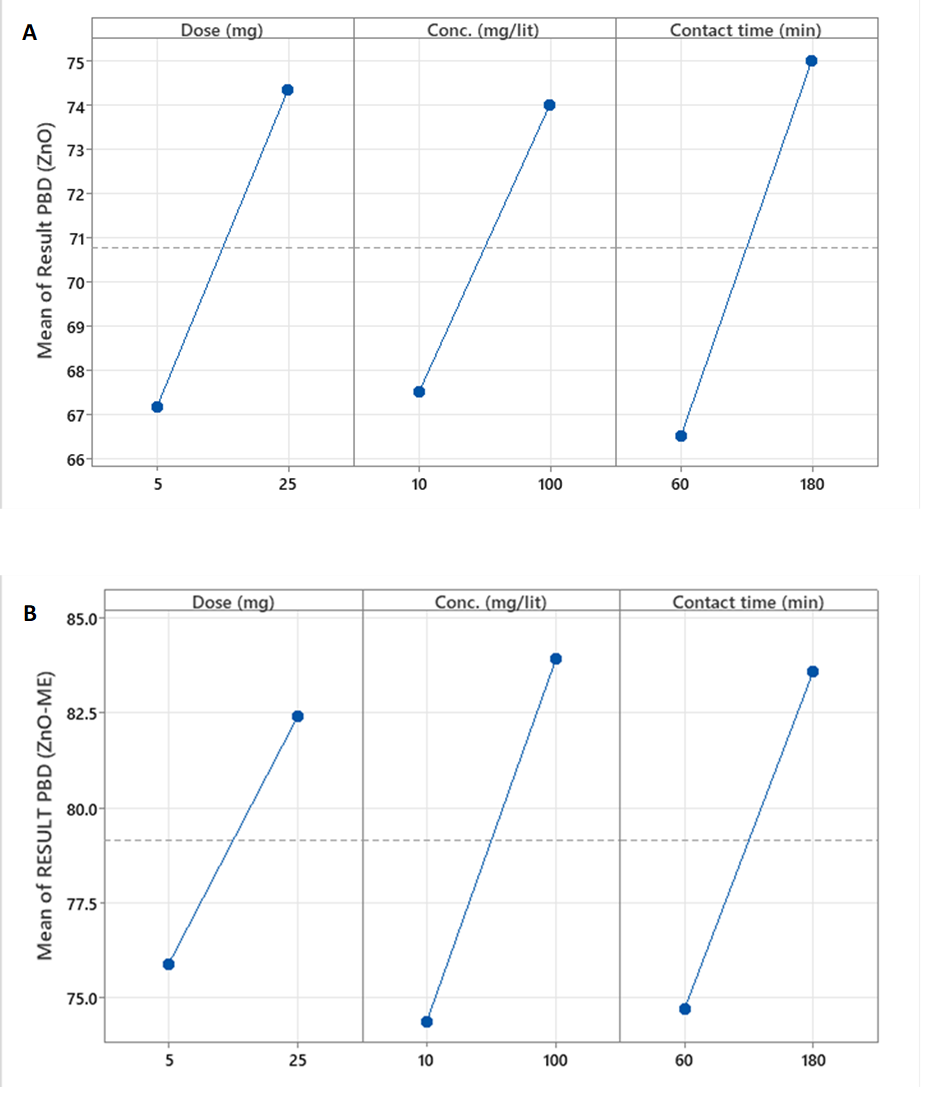


**Fig.S7** PBD- Main effects plot between the mean of results and parameters dose, initial dye conc. and contact time (A) ZnO and (B) ZnO-ME nanoparticles


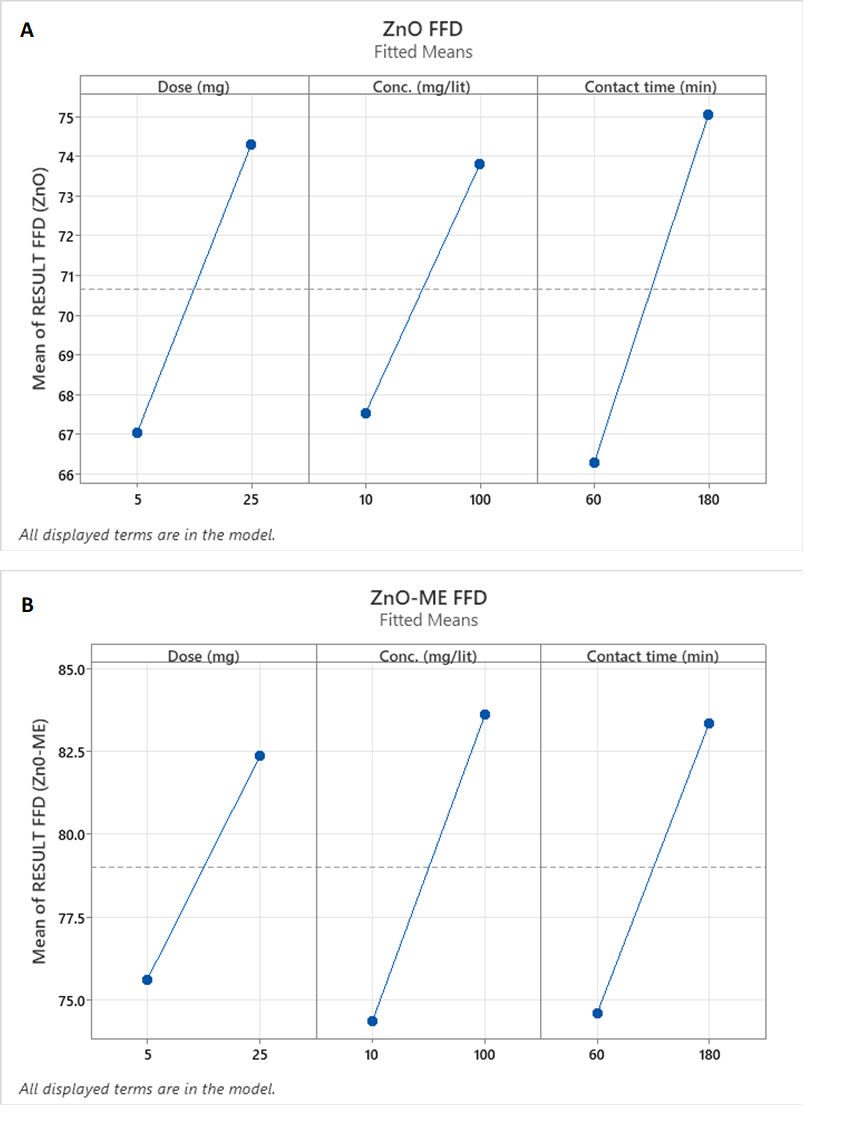


**Fig.S8** FFD- Main effects plot between the mean of results and parameters dose, initial dye conc. and contact time (A) ZnO and (B) ZnO-ME nanoparticles


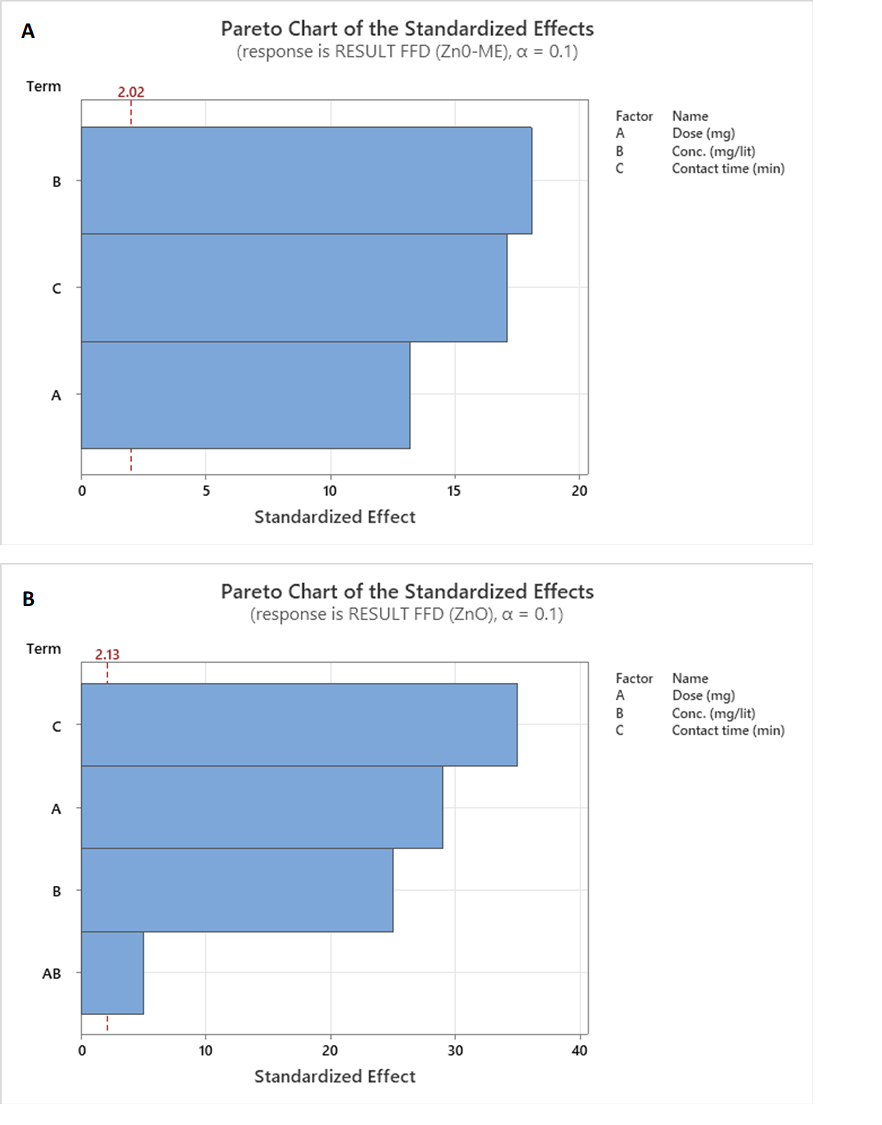


**Fig.S9** Pareto plots of ZnO and ZnO-ME nanoparticles between the parameters adsorbent dose, initial dye conc. and contact on the Y-axis and the standardized effect on the X-axis to show the effect of each parameter on the dye adsorption.


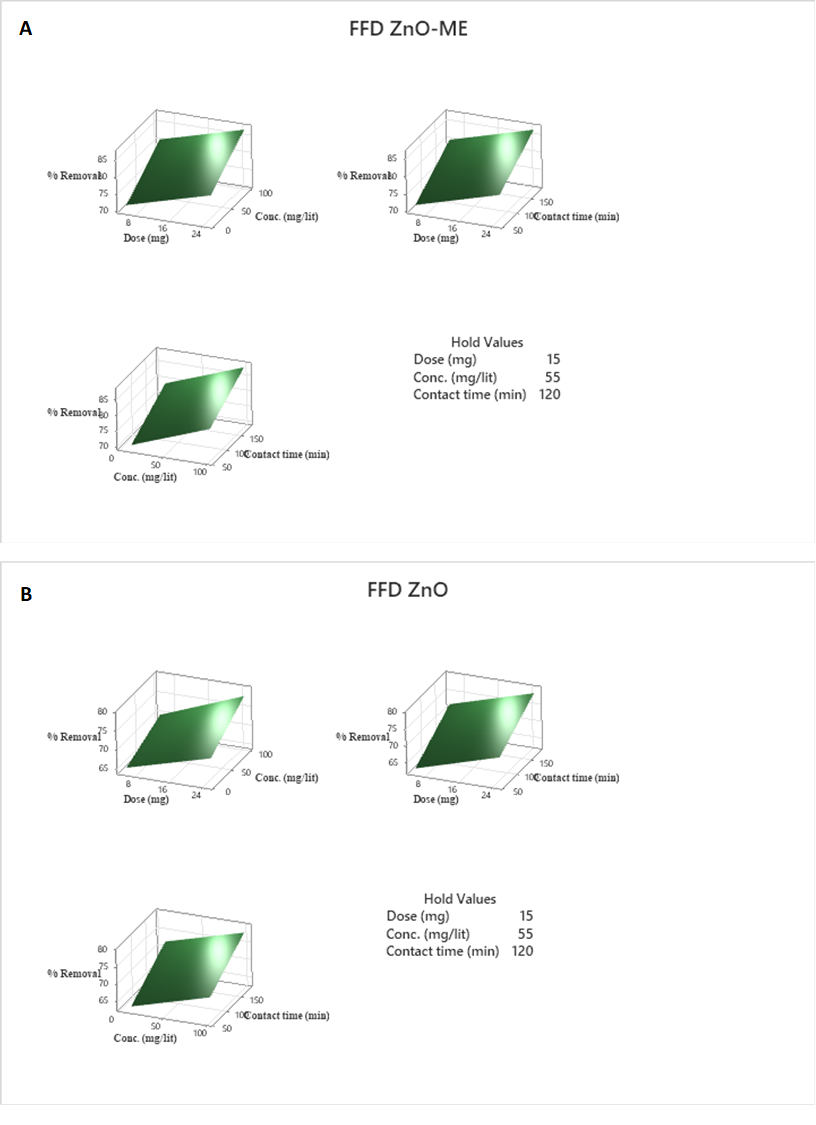


**Fig. S10** FFD-Three-dimensional response surfaces for sulfur black dye adsorption between three parameters, i.e., dose, initial dye conc. and contact time at hold values and percentage dye removal using (A) ZnO-ME and (B) ZnO nanoparticle


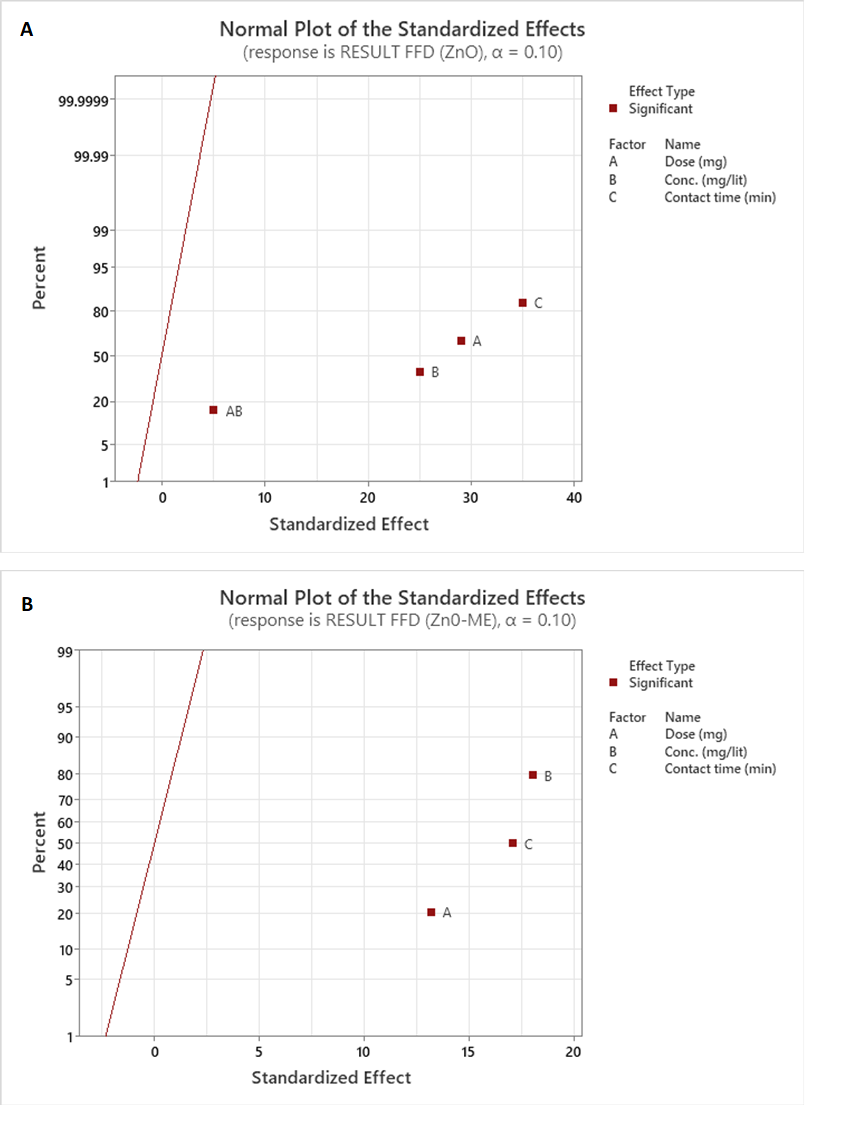


**Fig. S11** Normal plots of the standardized effects between the standardized effect on the X-axis and the percentage effect on the Y-axis for the experimental parameters, i.e., dose (A), initial dye conc. (B) and contact time (C) : (A) ZnO and (B) ZnO-ME nanoparticles


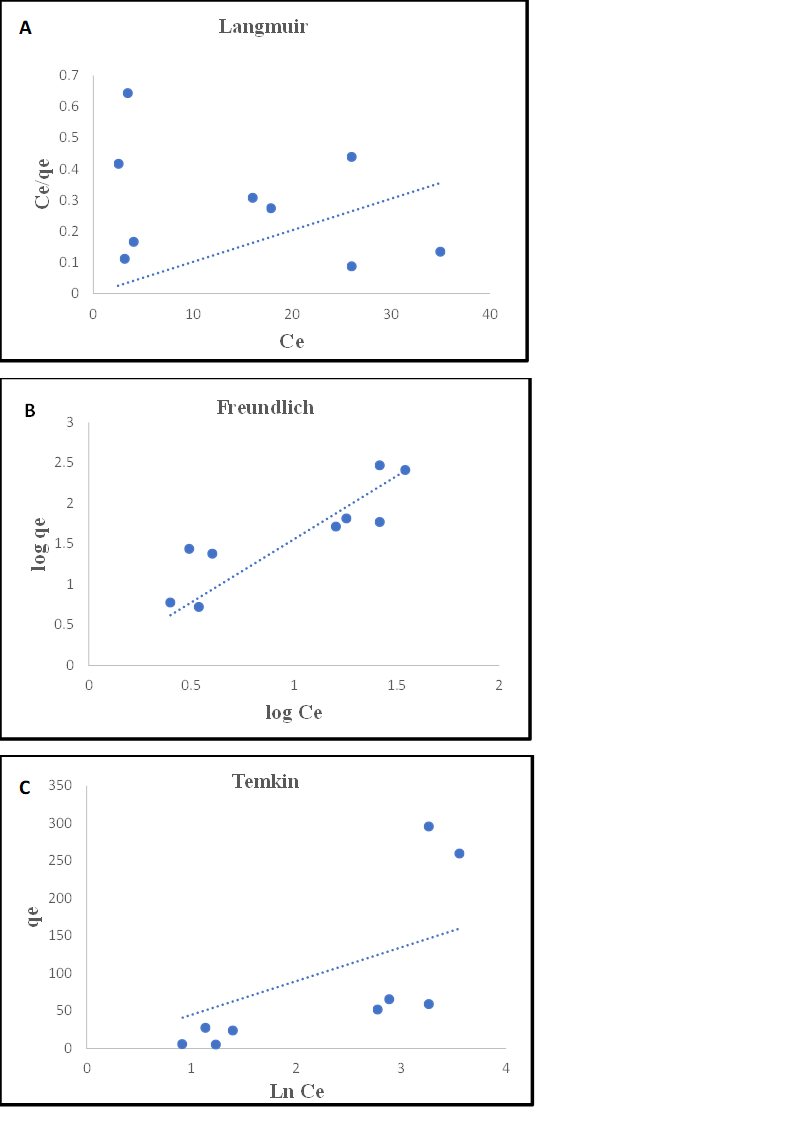


**Fig. S12** Adsorption isotherms models plots for ZnO nanoparticles: (A) Langmuir isotherm model, (B) Freundlich isotherm model and (C) Temkin isotherm model

**Fig. S13** Adsorption isothermal models plots for ZnO-ME nanoparticles: (A) Langmuir isotherm model, (B) Freundlich isotherm model and (C) Temkin isotherm model
